# Correction: Quantification of glucose-6-phosphate dehydrogenase activity by spectrophotometry: A systematic review and meta-analysis

**DOI:** 10.1371/journal.pmed.1003311

**Published:** 2020-07-24

**Authors:** Daniel A. Pfeffer, Benedikt Ley, Rosalind E. Howes, Patrick Adu, Mohammad Shafiul Alam, Pooja Bansil, Yap Boum, Marcelo Brito, Pimlak Charoenkwan, Archie Clements, Liwang Cui, Zeshuai Deng, Ochaka Julie Egesie, Fe Esperanza Espino, Michael E. von Fricken, Muzamil Mahdi Abdel Hamid, Yongshu He, Gisela Henriques, Wasif Ali Khan, Nimol Khim, Saorin Kim, Marcus Lacerda, Chanthap Lon, Asrat Hailu Mekuria, Didier Menard, Wuelton Monteiro, François Nosten, Nwe Nwe Oo, Sampa Pal, Duangdao Palasuwan, Sunil Parikh, Ayodhia Pitaloka Pasaribu, Jeanne Rini Poespoprodjo, David J. Price, Arantxa Roca-Feltrer, Michelle E. Roh, David L. Saunders, Michele D. Spring, Inge Sutanto, Kamala LeyThriemer, Thomas A. Weppelmann, Lorenz von Seidlein, Ari Winasti Satyagraha, Germana Bancone, Gonzalo J. Domingo, Ric N. Price

The name of author 40 was entered incorrectly. The correct name is Kamala Thriemer. Additionally, the affiliation listed for Thomas A. Weppelmann is incorrect. Affiliation 38 should therefore read: **38** Department of Internal Medicine, University of South Florida, Tampa, Florida, United States of America.
