# Supplementary material for: Quantification of glucose-6-phosphate dehydrogenase activity by spectrophotometry: A systematic review and meta-analysis
Source: PLoS Med. 2020 May 14;17(5):e1003084. doi: 10.1371/journal.pmed.1003084 (PMC7224463; doi:10.1371/journal.pmed.1003084)
Supplement: S1 File — (PDF) [file pmed.1003084.s001.pdf]

PRISMA 2009 Checklist

Section/topic	#	Checklist item	Reported on page #
**TITLE**			
Title	1	Identify the report as a systematic review, meta-analysis, or both.	**Page 1** : lines 1-2<br>**Title**: *Quantification of G6PD activity by spectrophotometry: A systematic review and meta-analysis*
**ABSTRACT**			
Structured summary	2	Provide a structured summary including, as applicable: background; objectives; data sources; study eligibility criteria, participants, and interventions; study appraisal and synthesis methods; results; limitations; conclusions and implications of key findings; systematic review registration number.	**Pages 2-3:**<br>Lines 88-123
**INTRODUCTION**			
Rationale	3	Describe the rationale for the review in the context of what is already known.	**Pages 6-7**:<br>**Rationale**: lines 181-202
Objectives	4	Provide an explicit statement of questions being addressed with reference to participants, interventions, comparisons, outcomes, and study design (PICOS).	**Pages 6-7**:<br>**Objectives**: lines 203-206
**METHODS**			
Protocol and registration	5	Indicate if a review protocol exists, if and where it can be accessed (e.g., Web address), and, if available, provide registration information including registration number.	**Page 7:**<br>Review registered at PROSPERO: CRD42019121414
Eligibility criteria	6	Specify study characteristics (e.g., PICOS, length of follow-up) and report characteristics (e.g., years considered, language, publication status) used as criteria for eligibility, giving rationale.	**Pages 7-8:**<br>Study characteristics and eligibility detailed in lines 209-227.
Information sources	7	Describe all information sources (e.g., databases with dates of coverage, contact with study authors to identify additional studies) in the search and date last searched.	**Page 7:**<br>Literature search detailed in lines 209-221.
Search	8	Present full electronic search strategy for at least one database, including any limits used, such that it could be repeated.	**Page 7**: lines 210-214<br>"Relevant studies involving individual-level spectrophotometric measurements of G6PD activity were identified via a PubMed search

PRISMA 2009 Checklist

			using the terms: *G6PD OR "glucose-6-phosphate dehydrogenase" OR "glucose 6 phosphate dehydrogenase") AND (quantitative OR spectrophot*"*
Study selection	9	State the process for selecting studies (i.e., screening, eligibility, included in systematic review, and, if applicable, included in the meta-analysis).	**Pages 7-8:** Study eligibility and exclusion detailed in lines 209-227.
Data collection process	10	Describe method of data extraction from reports (e.g., piloted forms, independently, in duplicate) and any processes for obtaining and confirming data from investigators.	**Page 7:** lines 219-220 "Authors were contacted via email and invited to contribute individual patient and quality control data."
Data items	11	List and define all variables for which data were sought (e.g., PICOS, funding sources) and any assumptions and simplifications made.	**Page 8:** Variables detailed in lines 228-231
Risk of bias in individual studies	12	Describe methods used for assessing risk of bias of individual studies (including specification of whether this was done at the study or outcome level), and how this information is to be used in any data synthesis.	**Page 8:** Risk of bias assessment described on lines 231-232: "Quality of included studies was assessed using an adapted form of the QUADAS-2 tool ([21]; **S1 File**)."
Summary measures	13	State the principal summary measures (e.g., risk ratio, difference in means).	**Pages 8-9:** Summary/ outcome measures detailed per-analysis in lines 243-265.
Synthesis of results	14	Describe the methods of handling data and combining results of studies, if done, including measures of consistency (e.g., I^2) for each meta-analysis.	**Page 8:** Data handling described in lines 228-232.

Section/topic	#	Checklist item	Reported on page #
Risk of bias across studies	15	Specify any assessment of risk of bias that may affect the cumulative evidence (e.g., publication bias, selective reporting within studies).	**Pages 9,18:** Exclusions based on risk of bias and discussion of influence due to possible bias in lines 250-251; 450-465 respectively.

Additional analyses	16	Describe methods of additional analyses (e.g., sensitivity or subgroup analyses, meta-regression), if done, indicating which were pre-specified.	**Pages 8-9:**<br>All analyses described on lines 239-265.
**RESULTS**			
Study selection	17	Give numbers of studies screened, assessed for eligibility, and included in the review, with reasons for exclusions at each stage, ideally with a flow diagram.	**Page 10:**<br>Lines 270-286; Figure 1
Study characteristics	18	For each study, present characteristics for which data were extracted (e.g., study size, PICOS, follow-up period) and provide the citations.	**S1 Table:**<br>Full table of study characteristics
Risk of bias within studies	19	Present data on risk of bias of each study and, if available, any outcome level assessment (see item 12).	**S2 Table:**<br>Risk of bias assessment for each study.
Results of individual studies	20	For all outcomes considered (benefits or harms), present, for each study: (a) simple summary data for each intervention group (b) effect estimates and confidence intervals, ideally with a forest plot.	a) **Summary data: Pages 11-12**, 15; Tables 1-3; Tables S3-4<br>b) **'Effect' estimates:** Figs 2-4 & S1; Figs S7-S18
Synthesis of results	21	Present results of each meta-analysis done, including confidence intervals and measures of consistency.	i) **Assay repeatability: Pages 11-12**; Table 2; Fig 2<br>ii) **Inter- and intra-laboratory variability: Pages 12-13:** Figs 3-4<br>iii) **Performance of Universal thresholds: Pages 13-15;** Table 3, Figs S7-18
Risk of bias across studies	22	Present results of any assessment of risk of bias across studies (see Item 15).	**Pages 17-18:** Bias due to specific assay choice, quality control or participant characteristics discussed in lines 432-465
Additional analysis	23	Give results of additional analyses, if done (e.g., sensitivity or subgroup analyses, meta-regression [see Item 16]).	**Pages 14-15:**<br>Sensitivity analyses lines 369-274; Fig S4-S6; S13-S18; Table S3-S4
**DISCUSSION**			
Summary of evidence	24	Summarize the main findings including the strength of evidence for each main outcome; consider their relevance to key groups (e.g., healthcare providers, users, and policy makers).	**Pages 16-17:**<br>Lines 397-431

Limitations	25	Discuss limitations at study and outcome level (e.g., risk of bias), and at review-level (e.g., incomplete retrieval of identified research, reporting bias).	**Pages 17-18:** Lines 432-465
Conclusions	26	Provide a general interpretation of the results in the context of other evidence, and implications for future research.	**Page 19:** Lines 467-480
**FUNDING**			
Funding	27	Describe sources of funding for the systematic review and other support (e.g., supply of data); role of funders for the systematic review.	**Page 19:** Lines 486-488

From: Moher D, Liberati A, Tetzlaff J, Altman DG, The PRISMA Group (2009). Preferred Reporting Items for Systematic Reviews and Meta-Analyses: The PRISMA Statement. PLoS Med 6(7): e1000097. doi:10.1371/journal.pmed1000097

For more information, visit: **www.prisma-statement.org**.
