# Supplementary material for: Quantification of glucose-6-phosphate dehydrogenase activity by spectrophotometry: A systematic review and meta-analysis
Source: PLoS Med. 2020 May 14;17(5):e1003084. doi: 10.1371/journal.pmed.1003084 (PMC7224463; doi:10.1371/journal.pmed.1003084)
Supplement: S2 File — QUADAS-2, Quality Assessment of Diagnostic Accuracy Studies-2. (PDF) [file pmed.1003084.s002.pdf]

		Yes	No	Unclear
**Risk of Bias**				
Patient Selection	**1. Was a consecutive or random sample of patients enrolled?**	☐	☐	☐
	**2. Was a case control design avoided?**	☐	☐	☐
	3. Did the study avoid inappropriate exclusions?	☐	☐	☐
Spectrophotometry	**4. Is the spectrophotometry assay appropriate to correctly classify the target condition?**	☐	☐	☐
	5. Were replicate measures performed?	☐	☐	☐
	**6. Were quality control measurements included?**	☐	☐	☐
Flow and Timing	7. Was there an appropriate interval between sample collection and spectrophotometry?	☐	☐	☐
	8. Did all patients receive the same spectrophotometry assay?	☐	☐	☐
	9. Were all patients included in the analysis?	☐	☐	☐
**Applicability**				
Patient Selection	10. Was the target population applicable to the review?	☐	☐	☐
Spectrophotometry	11. Was the spectrophotometry assay applicable to the review?	☐	☐	☐

bold font = 'major' sub-question; normal font = 'minor' sub-question

Overall quality classifications:

Low = 'Yes' to all sub-questions with up to 1 'unclear' for minor sub-questions.
Possible = 'No' to only one minor sub-question.
High = 'No' to one or more major sub-question(s).
Unclear = 'Unclear' to at least one major sub-question or multiple minor sub-questions.
