# Supplementary material for: Quantification of glucose-6-phosphate dehydrogenase activity by spectrophotometry: A systematic review and meta-analysis
Source: PLoS Med. 2020 May 14;17(5):e1003084. doi: 10.1371/journal.pmed.1003084 (PMC7224463; doi:10.1371/journal.pmed.1003084)
Supplement: S3 File — (PDF) [file pmed.1003084.s003.pdf]

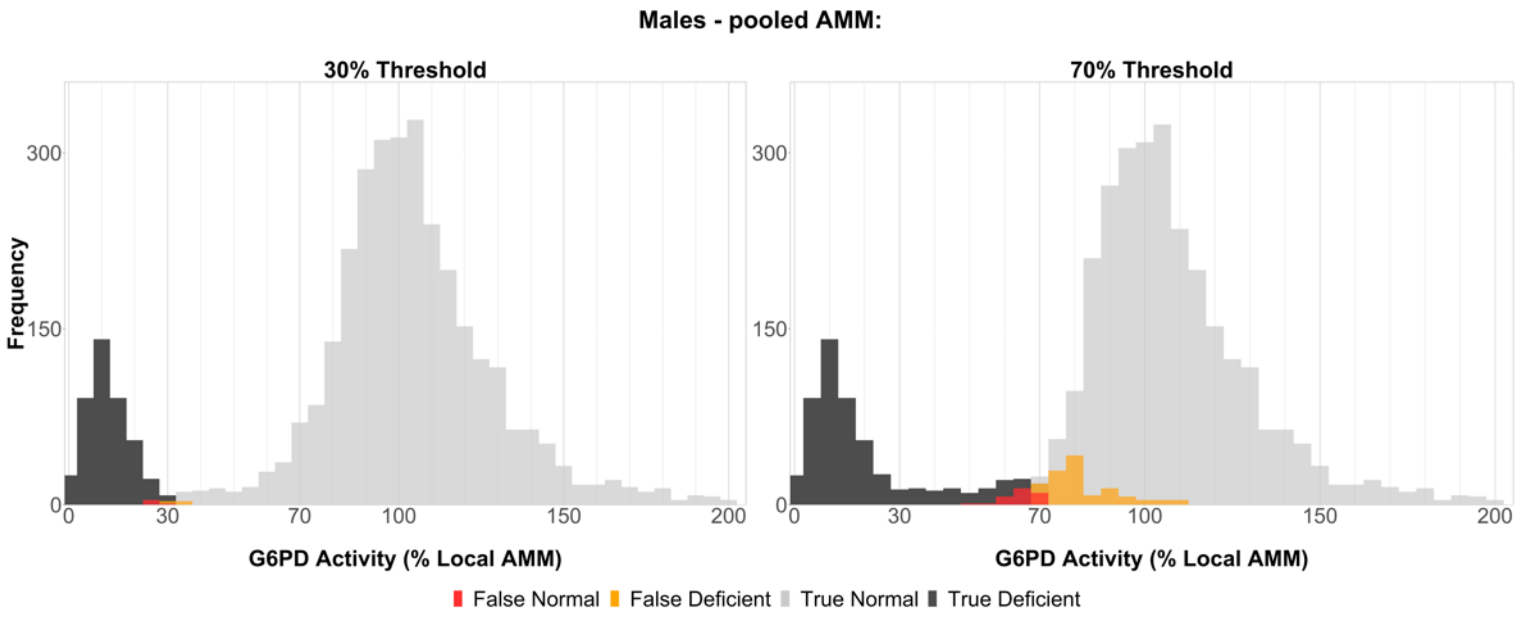

S2 Fig. Universal diagnostic classifications by G6PD activity – males, using pooled AMM
Relative G6PD activity is shown (x-axis; normalized to site-specific AMM) for individuals falling into each diagnostic category, as depicted by coloured bars (false normal = red, false deficient = orange, true normal = light grey, true deficient = black). Diagnoses at both the 30% threshold (left; 5 FN, 6 FD, 3,072 TN, 433 TD) and 70% threshold (right; 33 FN, 130 FD, 2,786 TN, 567 TD) are shown. All males tested with trinity without malaria infection are included, except 31 individuals with G6PD activity >200% local AMM (n = 3,485).

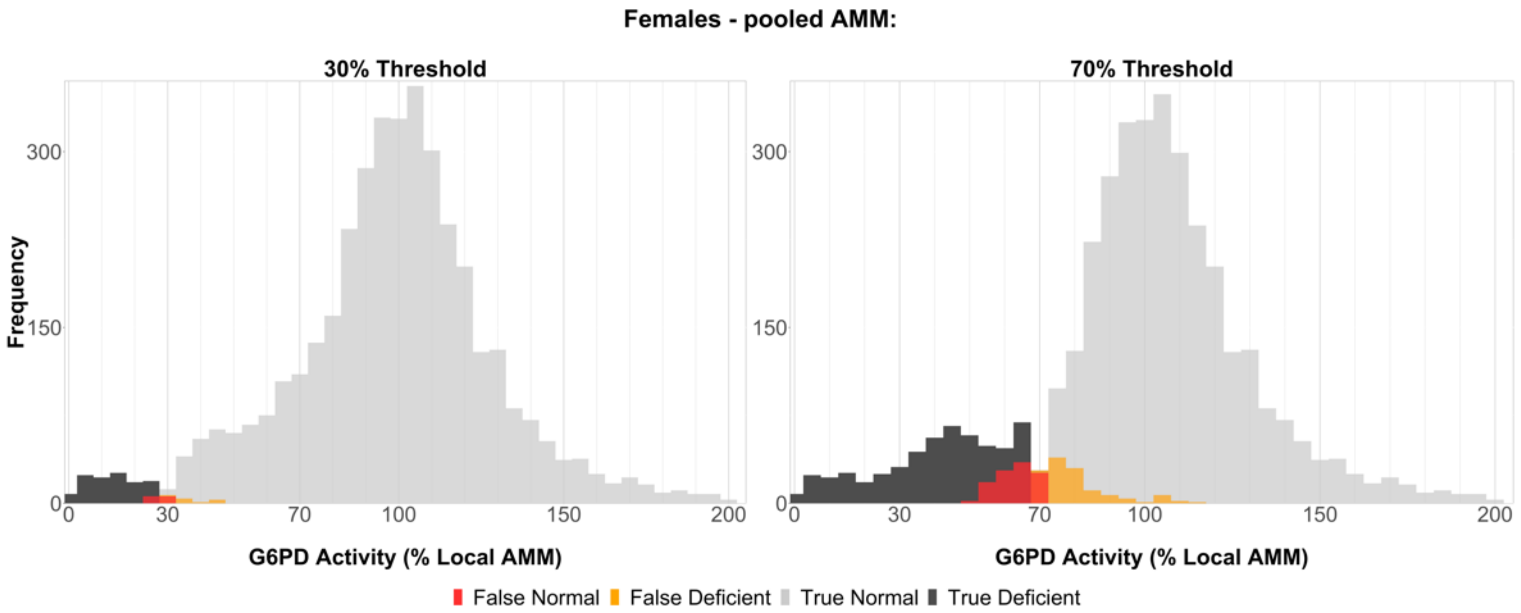

S3 Fig. Universal diagnostic classifications by G6PD activity – females, using pooled AMM
Relative G6PD activity is shown (x-axis; normalized to site-specific AMM) for individuals falling into each diagnostic category, as depicted by coloured bars (false normal = red, false deficient = orange, true normal = light grey, true deficient = black). Diagnoses at both the 30% threshold (left; 12 FN, 15 FD, 3,854 TN, 123 TD) and 70% threshold (right; 109 FN, 130 FD, 3,194 TN, 571 TD) are shown. All males tested with trinity without malaria infection are included, except 38 individuals with G6PD activity >200% local AMM (n = 3,966).

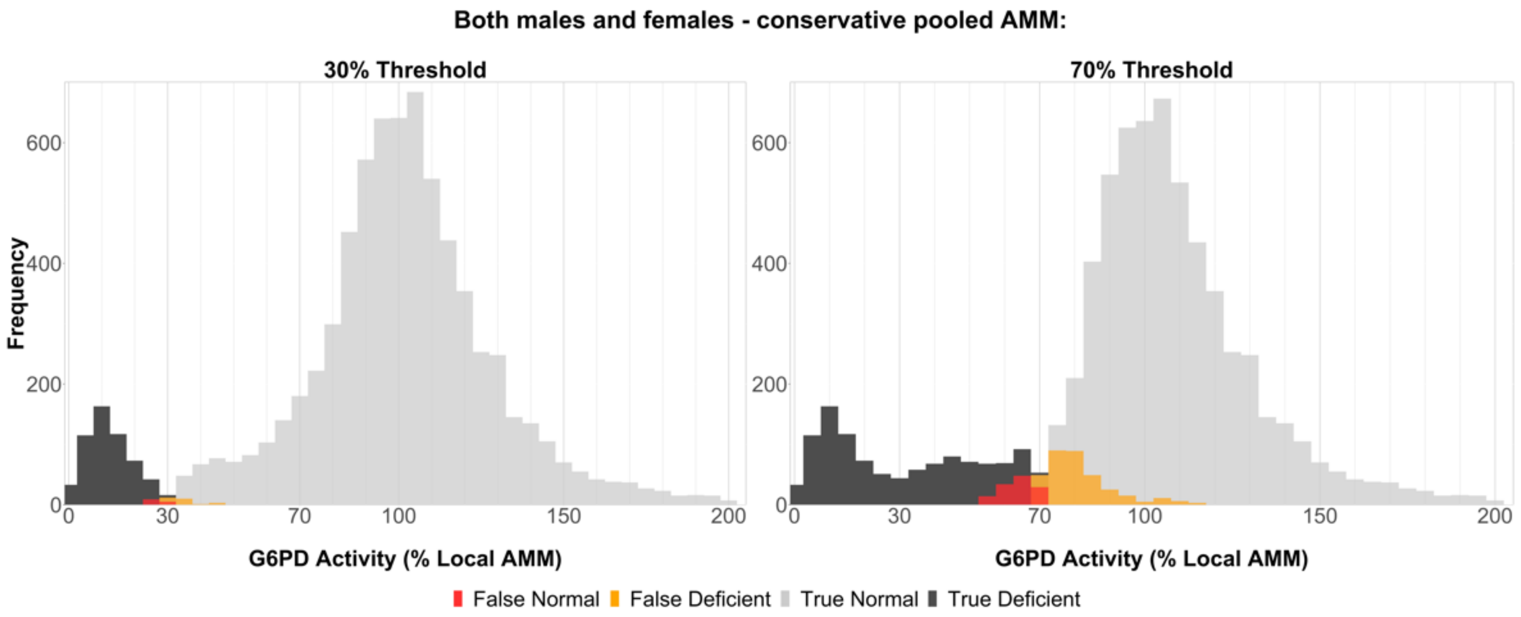

S4 Fig. Universal diagnostic classifications by G6PD activity – both males and females, using conservative pooled AMM
Relative G6PD activity is shown (x-axis; normalized to site-specific AMM) for individuals falling into each diagnostic category, as depicted by coloured bars (false normal = red, false deficient = orange, true normal = light grey, true deficient = black). Diagnoses at both the 30% threshold (left; 14 FN, 26 FD, 6,921 TN, 559 TD) and 70% threshold (right; 125 FN, 342 FD, 5,898 TN, 1,155 TD) are shown. All individuals tested with trinity without malaria infection are included, except 69 individuals with G6PD activity >200% local AMM (n = 7,451).

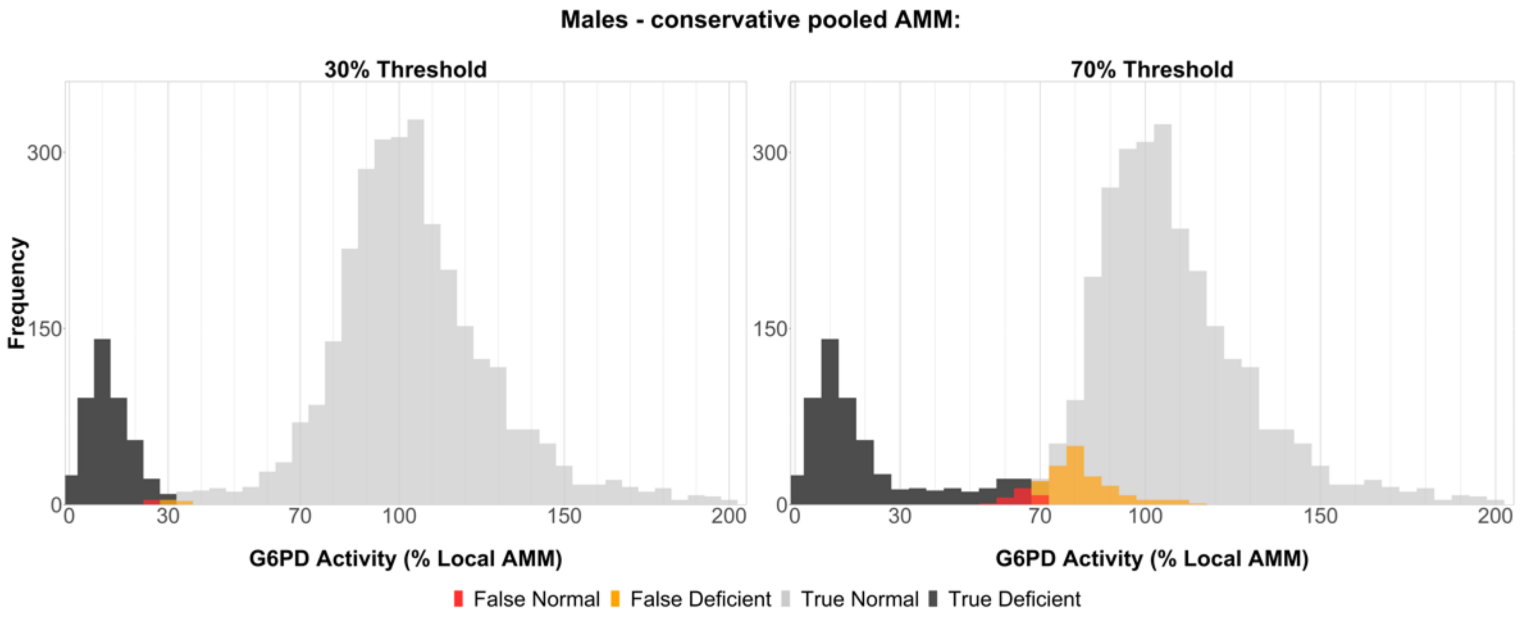

S5 Fig. Universal diagnostic classifications by G6PD activity – males, using conservative pooled AMM
Relative G6PD activity is shown (x-axis; normalized to site-specific AMM) for individuals falling into each diagnostic category, as depicted by coloured bars (false normal = red, false deficient = orange, true normal = light grey, true deficient = black). Diagnoses at both the 30% threshold (left; 4 FN, 7 FD, 3,071 TN, 434 TD) and 70% threshold (right; 29 FN, 164 FD, 2,752 TN, 571 TD) are shown. All males tested with trinity without malaria infection are included, except 31 individuals with G6PD activity >200% local AMM (n = 3,485).

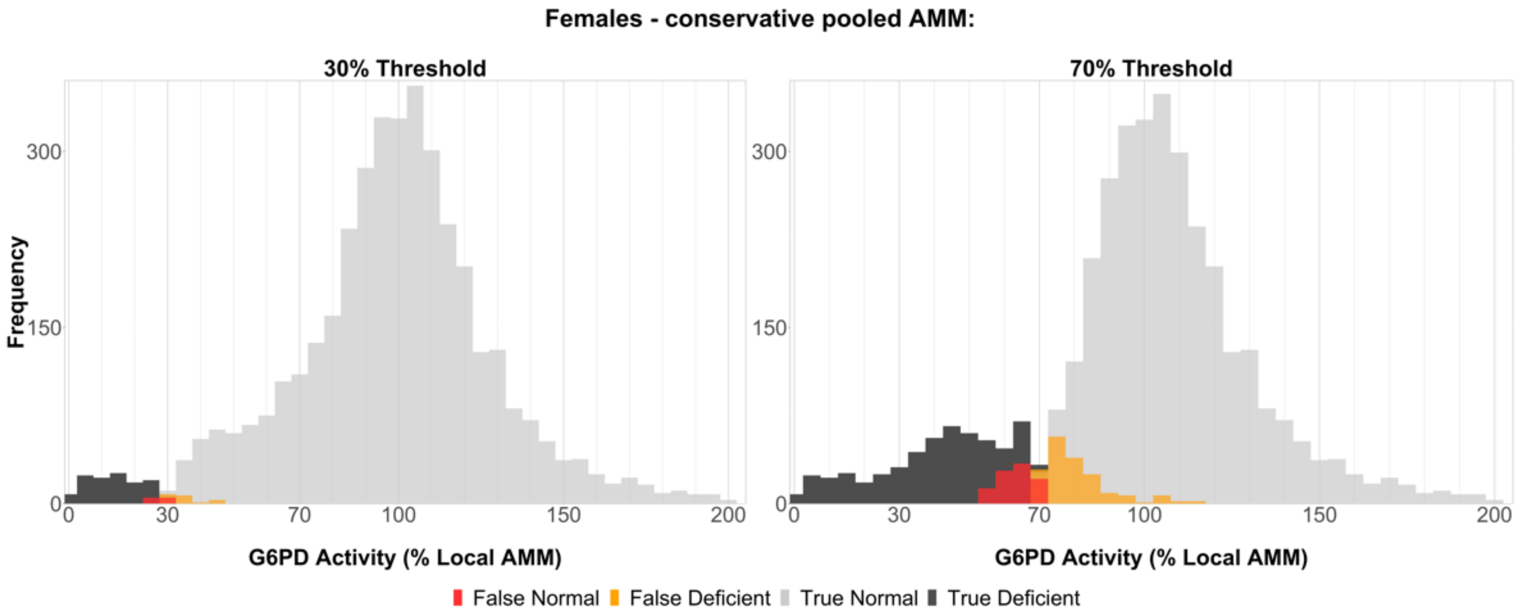

S6 Fig. Universal diagnostic classifications by G6PD activity – females, using conservative pooled AMM
Relative G6PD activity is shown (x-axis; normalized to site-specific AMM) for individuals falling into each diagnostic category, as depicted by coloured bars (false normal = red, false deficient = orange, true normal = light grey, true deficient = black). Diagnoses at both the 30% threshold (left; 10 FN, 19 FD, 3,850 TN, 125 TD) and 70% threshold (right; 96 FN, 178 FD, 3,146 TN, 584 TD) are shown. All males tested with trinity without malaria infection are included, except 38 individuals with G6PD activity >200% local AMM (n = 3,966).

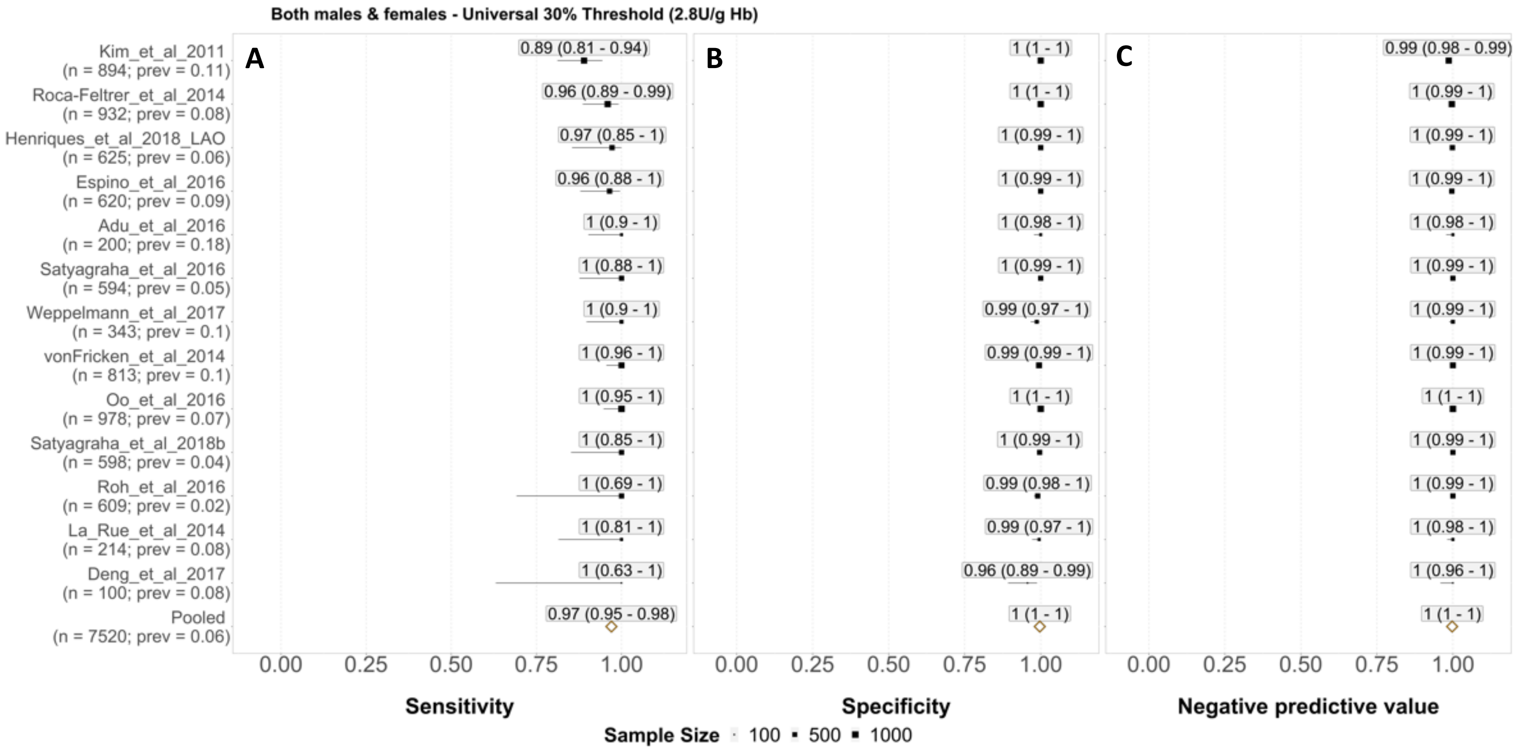

S7 Fig. Study-wise diagnostic performance of a pooled universal 30% enzyme activity threshold – both males and females. Study-wise sensitivity (**Panel A**), specificity (**Panel B**), & negative predictive value (**Panel C**) were calculated with 'exact' binomial confidence limits using site specific local AMM as the reference in each case. The universal 30% threshold used corresponds to 2.8 U/g Hb, as derived from a universal AMM (9.4 U/g Hb) calculated using all male samples without malaria tested using Trinity. Sample size and prevalence of G6PD deficiency (enzyme activity <30% local AMM) are indicated for each study on the y-axis. Points are sized according to sample size. Data were included from 7,520 males and females tested by Trinity.

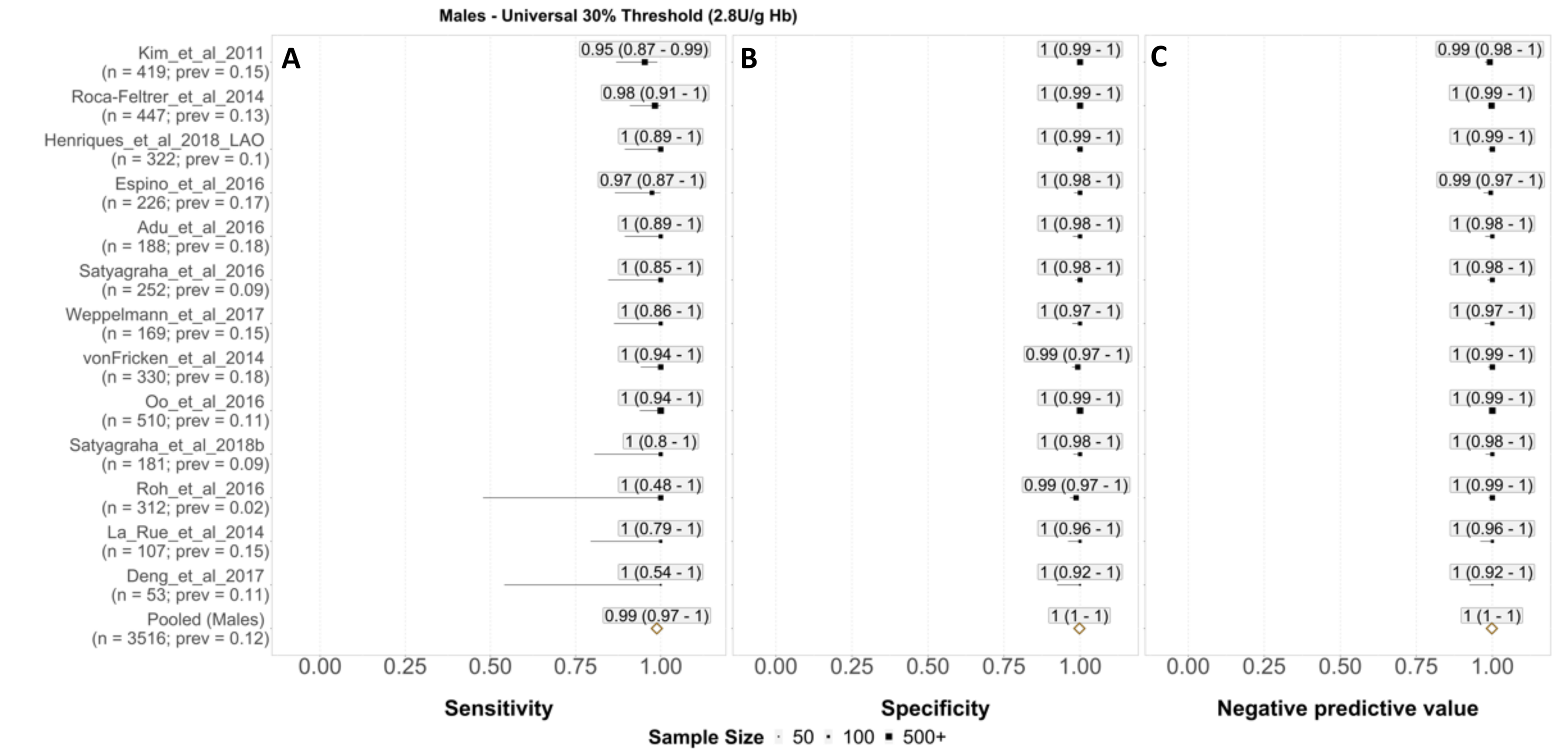

S8 Fig. Study-wise diagnostic performance of a pooled universal 30% enzyme activity threshold – males.
Study-wise sensitivity (**Panel A**), specificity (**Panel B**), & negative predictive value (**Panel C**) were calculated with 'exact' binomial confidence limits using site specific local AMM as the reference in each case. The universal 30% threshold used corresponds to 2.8 U/g Hb, as derived from a universal AMM (9.4 U/g Hb) calculated using all male samples without malaria tested using Trinity. Sample size and prevalence of G6PD deficiency (enzyme activity <30% local AMM) are indicated for each study on the y-axis. Points are sized according to sample size. Data were included from 3,516 males tested by Trinity.

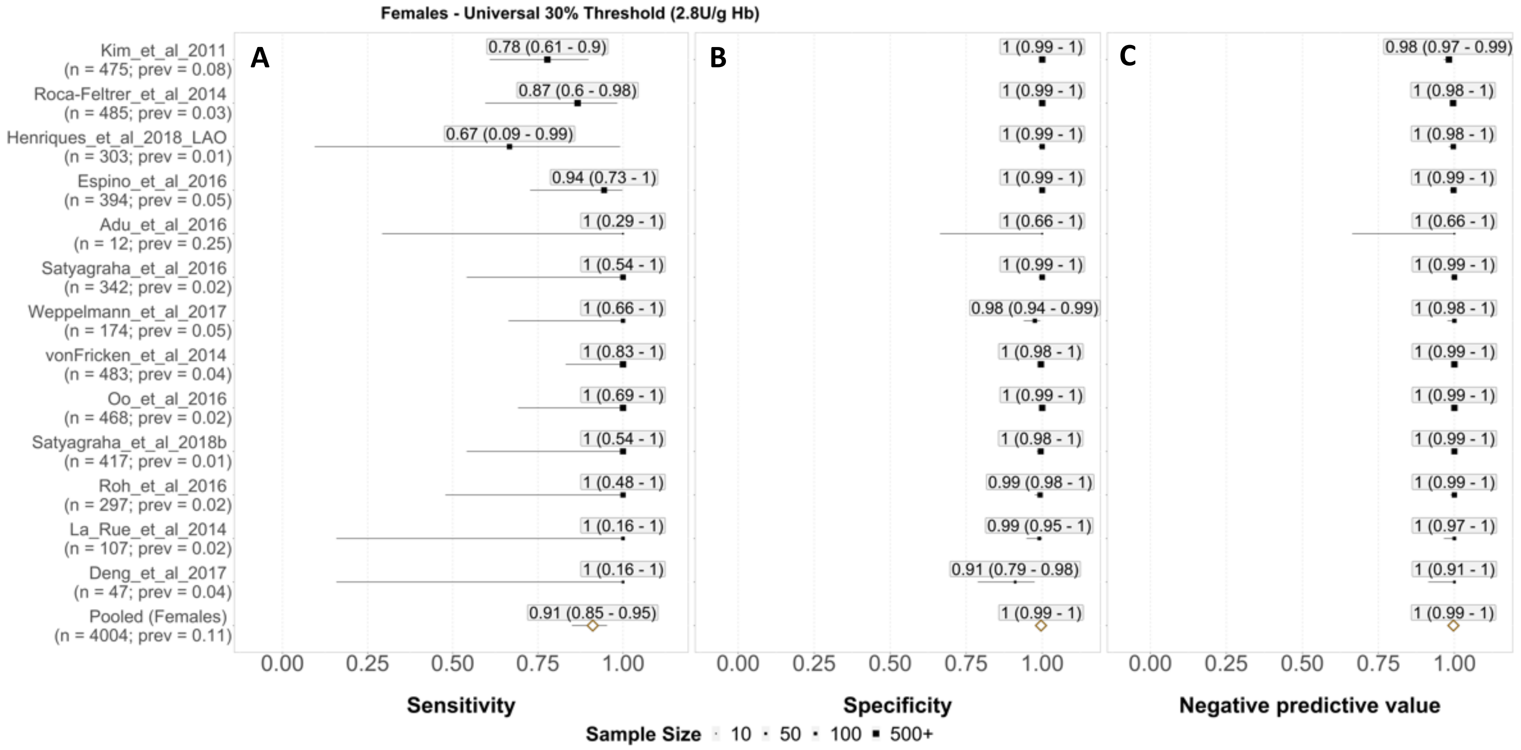

S9 Fig. Study-wise diagnostic performance of a pooled universal 30% enzyme activity threshold – females.
Study-wise sensitivity (**Panel A**), specificity (**Panel B**), & negative predictive value (**Panel C**) were calculated with 'exact' binomial confidence limits using site specific local AMM as the reference in each case. The universal 30% threshold used corresponds to 2.8 U/g Hb, as derived from a universal AMM (9.4 U/g Hb) calculated using all male samples without malaria tested using Trinity. Sample size and prevalence of G6PD deficiency (enzyme activity <30% local AMM) are indicated for each study on the y-axis. Points are sized according to sample size. Data were included from 4,004 females tested by Trinity.

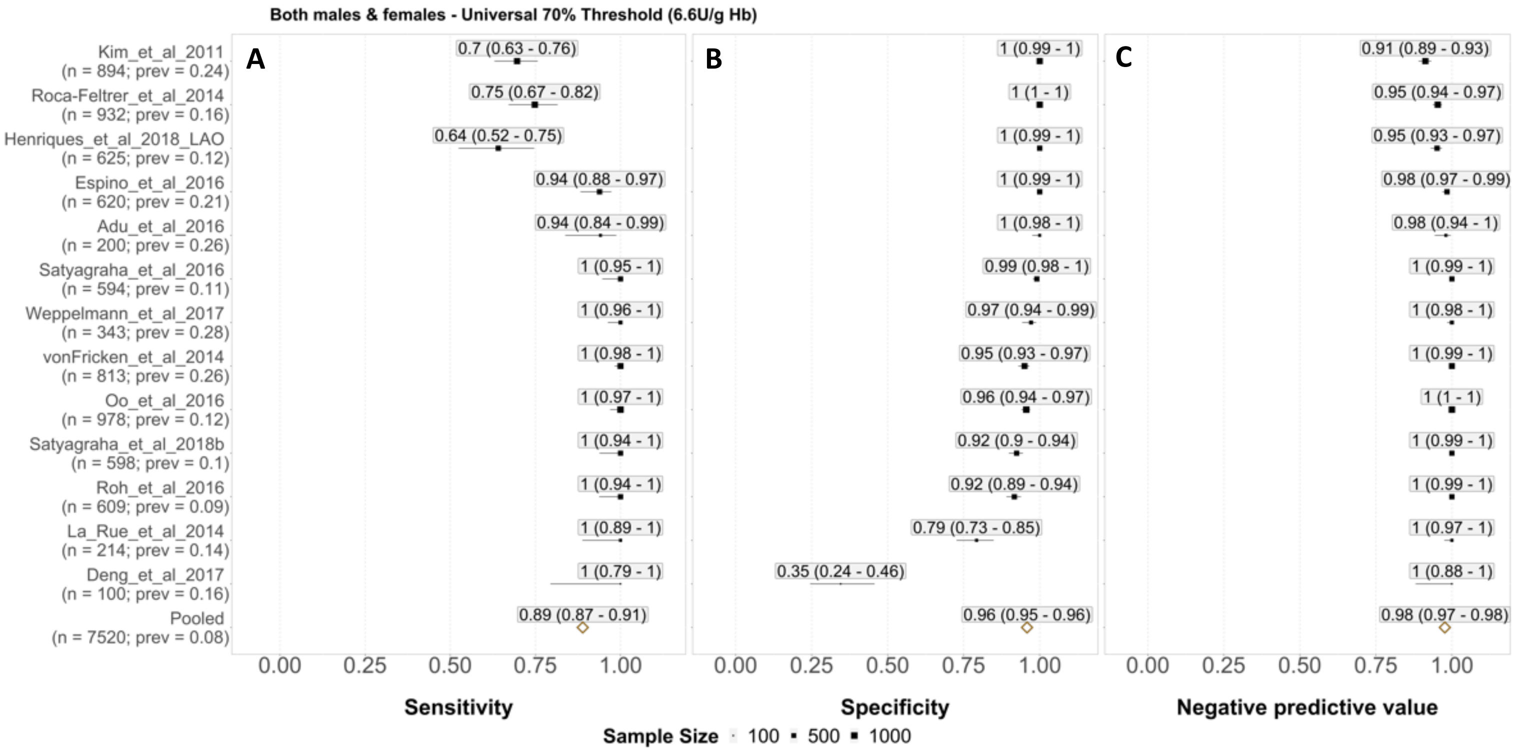

S10 Fig. Study-wise diagnostic performance of a pooled universal 70% enzyme activity threshold – both males and females. Study-wise sensitivity (**Panel A**), specificity (**Panel B**), & negative predictive value (**Panel C**) were calculated with 'exact' binomial confidence limits using site specific local AMM as the reference. The universal 70% threshold used corresponds to 6.6 U/g Hb, as derived from a universal AMM (9.4 U/g Hb) calculated from male samples without malaria tested using Trinity. Sample size and prevalence of severe or intermediate G6PD deficiency (enzyme activity <70% local AMM) are indicated for each study on the y-axis. Points are sized according to sample size. Data were included from 7,520 males and females tested by Trinity.

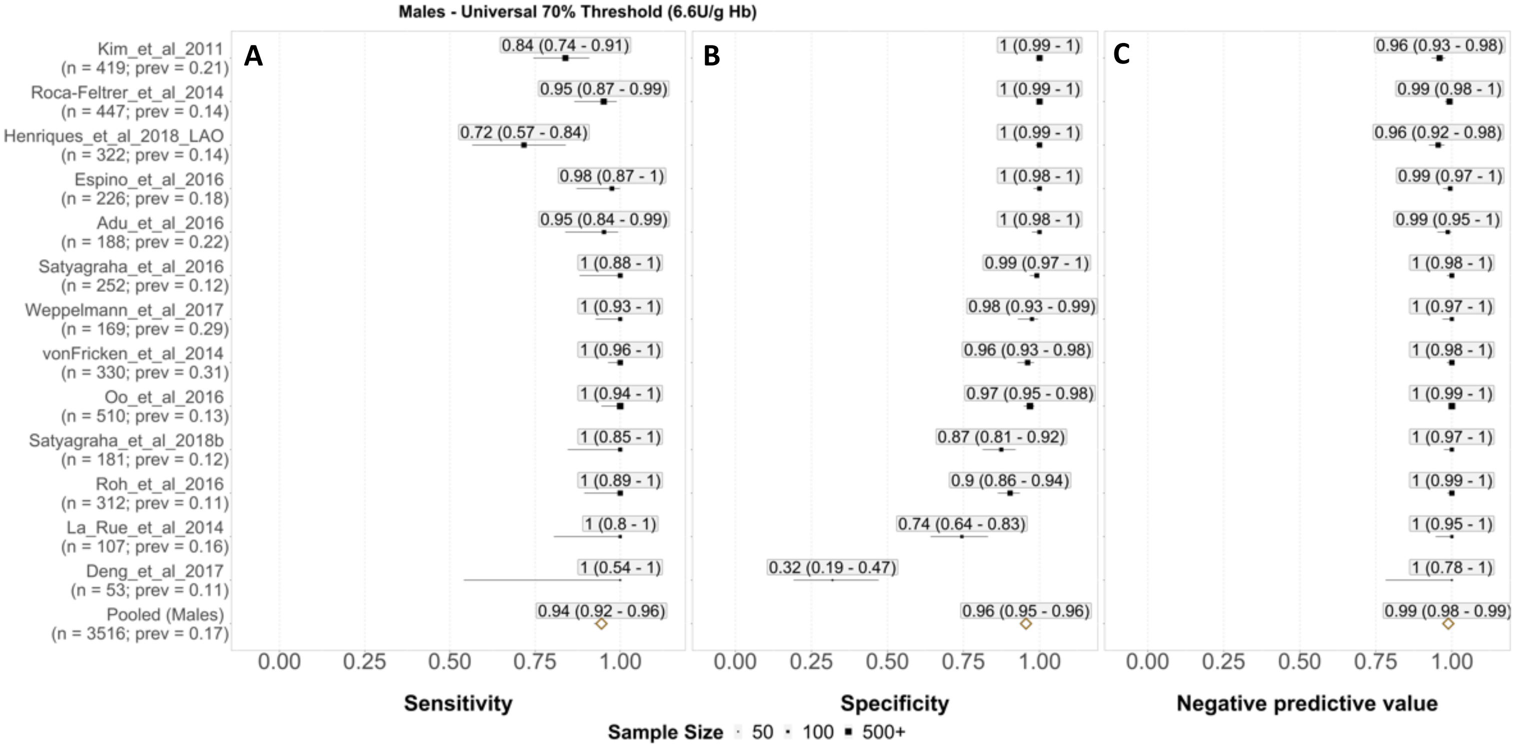

S11 Fig. Study-wise diagnostic performance of a pooled universal 70% enzyme activity threshold – males.
Study-wise sensitivity (**Panel A**), specificity (**Panel B**), & negative predictive value (**Panel C**) were calculated with 'exact' binomial confidence limits using site specific local AMM as the reference. The universal 70% threshold used corresponds to 6.6 U/g Hb, as derived from a universal AMM (9.4 U/g Hb) calculated from male samples without malaria tested using Trinity. Sample size and prevalence of severe or intermediate G6PD deficiency (enzyme activity <70% local AMM) are indicated for each study on the y-axis. Points are sized according to sample size. Data were included from 3,516 males tested by Trinity.

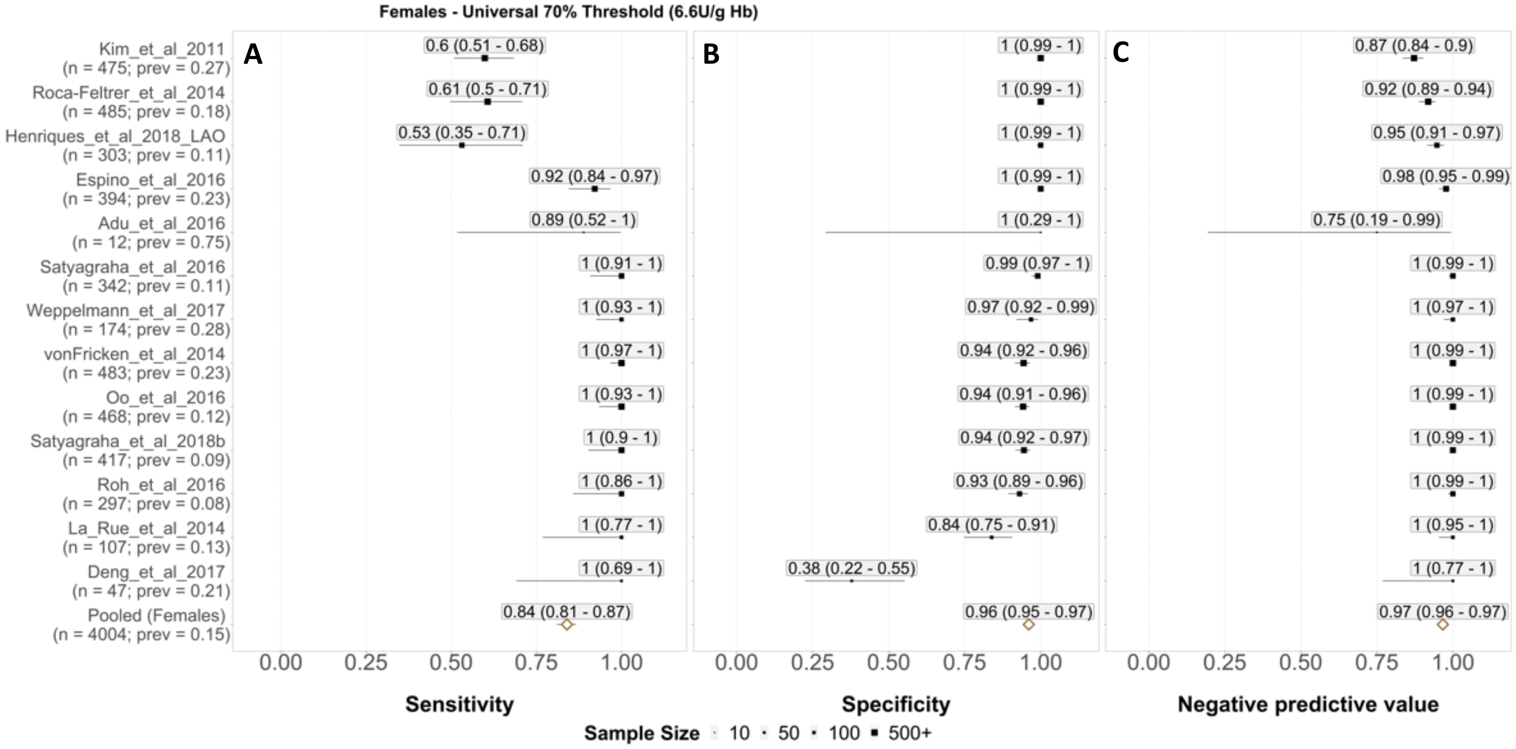

S12 Fig. Study-wise diagnostic performance of a pooled universal 70% enzyme activity threshold – females.
Study-wise sensitivity (**Panel A**), specificity (**Panel B**), & negative predictive value (**Panel C**) were calculated with 'exact' binomial confidence limits using site specific local AMM as the reference. The universal 70% threshold used corresponds to 6.6 U/g Hb, as derived from a universal AMM (9.4 U/g Hb) calculated from male samples without malaria tested using Trinity. Sample size and prevalence of severe or intermediate G6PD deficiency (enzyme activity <70% local AMM) are indicated for each study on the y-axis. Points are sized according to sample size. Data were included from 7,520 males and females tested by Trinity.

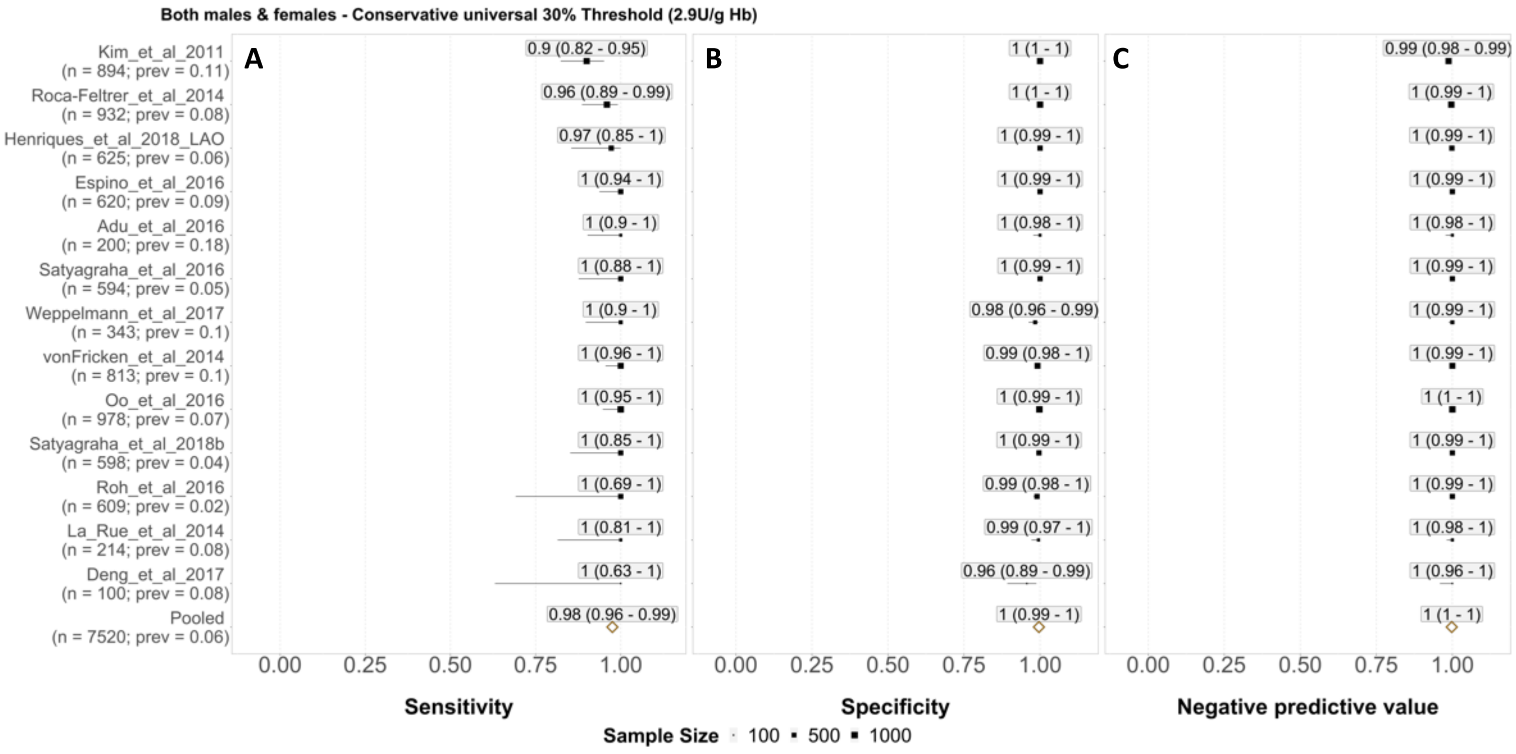

S13 Fig. Study-wise diagnostic performance of a conservative universal 30% enzyme activity threshold – both males and females.

Study-wise sensitivity (**Panel A**), specificity (**Panel B**), & negative predictive value (**Panel C**) were calculated with 'exact' binomial confidence limits using site specific local AMM as the reference in each case. The universal 30% threshold used corresponds to 2.9 U/g Hb, as derived from a conservative universal AMM (9.7 U/g Hb) – see methods for definition. Sample size and prevalence of G6PD deficiency (enzyme activity <30% local AMM) are indicated for each study on the y-axis. Points are sized according to sample size. Data were included from 7,520 males and females tested by Trinity.

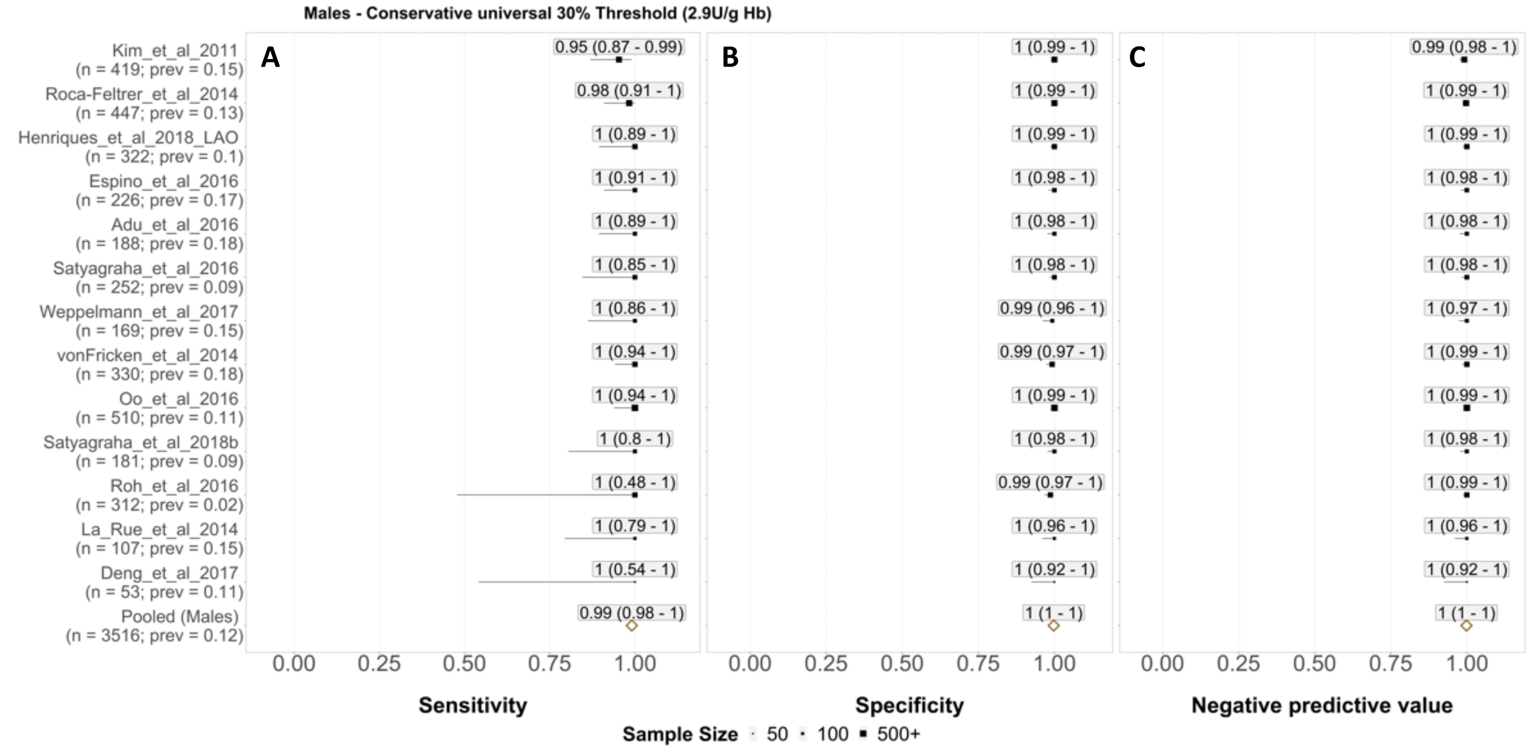

S14 Fig. Study-wise diagnostic performance of a pooled universal 30% enzyme activity threshold – males.
Study-wise sensitivity (**Panel A**), specificity (**Panel B**), & negative predictive value (**Panel C**) were calculated with 'exact' binomial confidence limits using site specific local AMM as the reference in each case. The universal 30% threshold used corresponds to 2.9 U/g Hb, as derived from a conservative universal AMM (9.7 U/g Hb) – see methods for definition. Sample size and prevalence of G6PD deficiency (enzyme activity <30% local AMM) are indicated for each study on the y-axis. Points are sized according to sample size. Data were included from 3,516 males tested by Trinity.

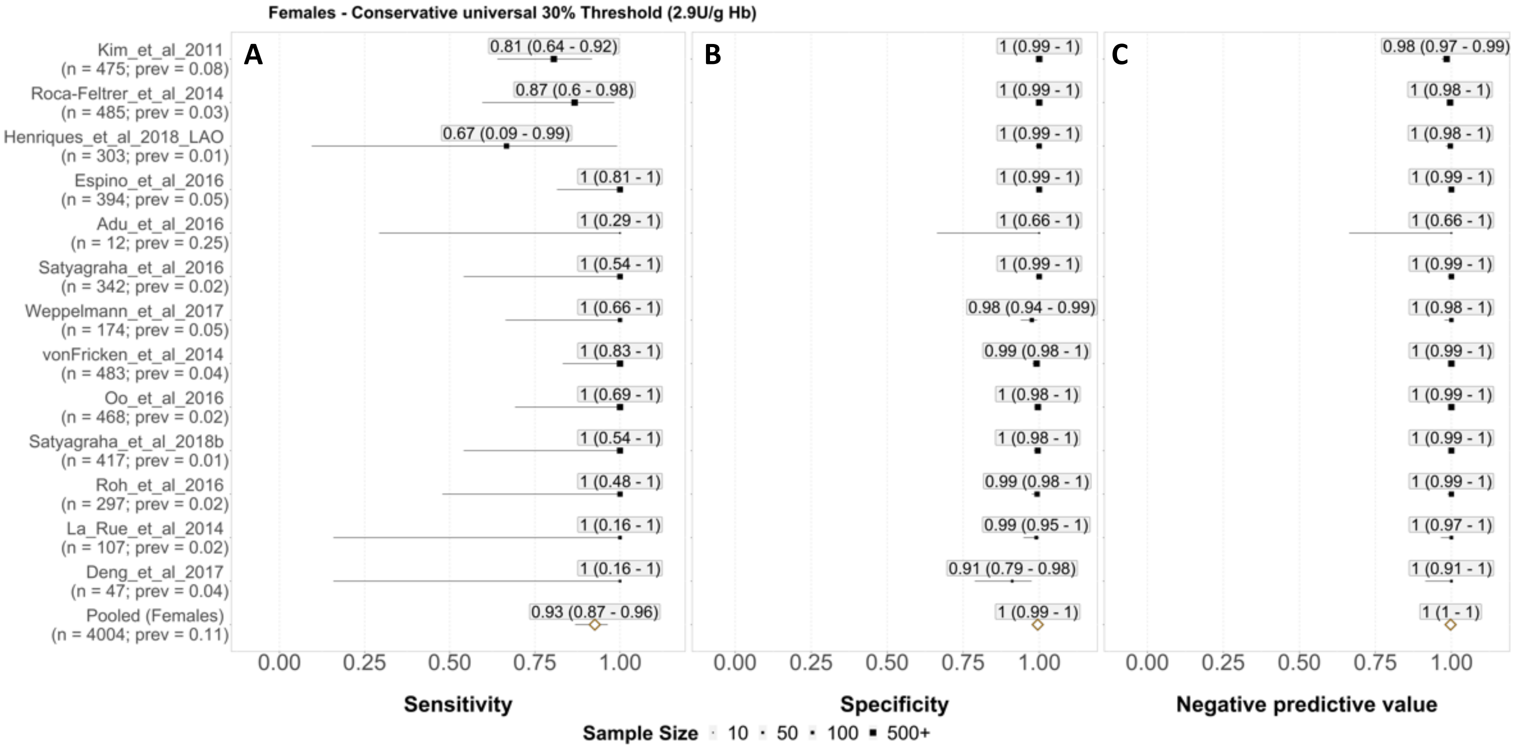

S15 Fig. Study-wise diagnostic performance of a conservative universal 30% enzyme activity threshold – females.
Study-wise sensitivity (**Panel A**), specificity (**Panel B**), & negative predictive value (**Panel C**) were calculated with 'exact' binomial confidence limits using site specific local AMM as the reference in each case. The universal 30% threshold used corresponds to 2.9 U/g Hb, as derived from a conservative universal AMM (9.7 U/g Hb) – see methods for definition. Sample size and prevalence of G6PD deficiency (enzyme activity <30% local AMM) are indicated for each study on the y-axis. Points are sized according to sample size. Data were included from 4,004 females tested by Trinity.

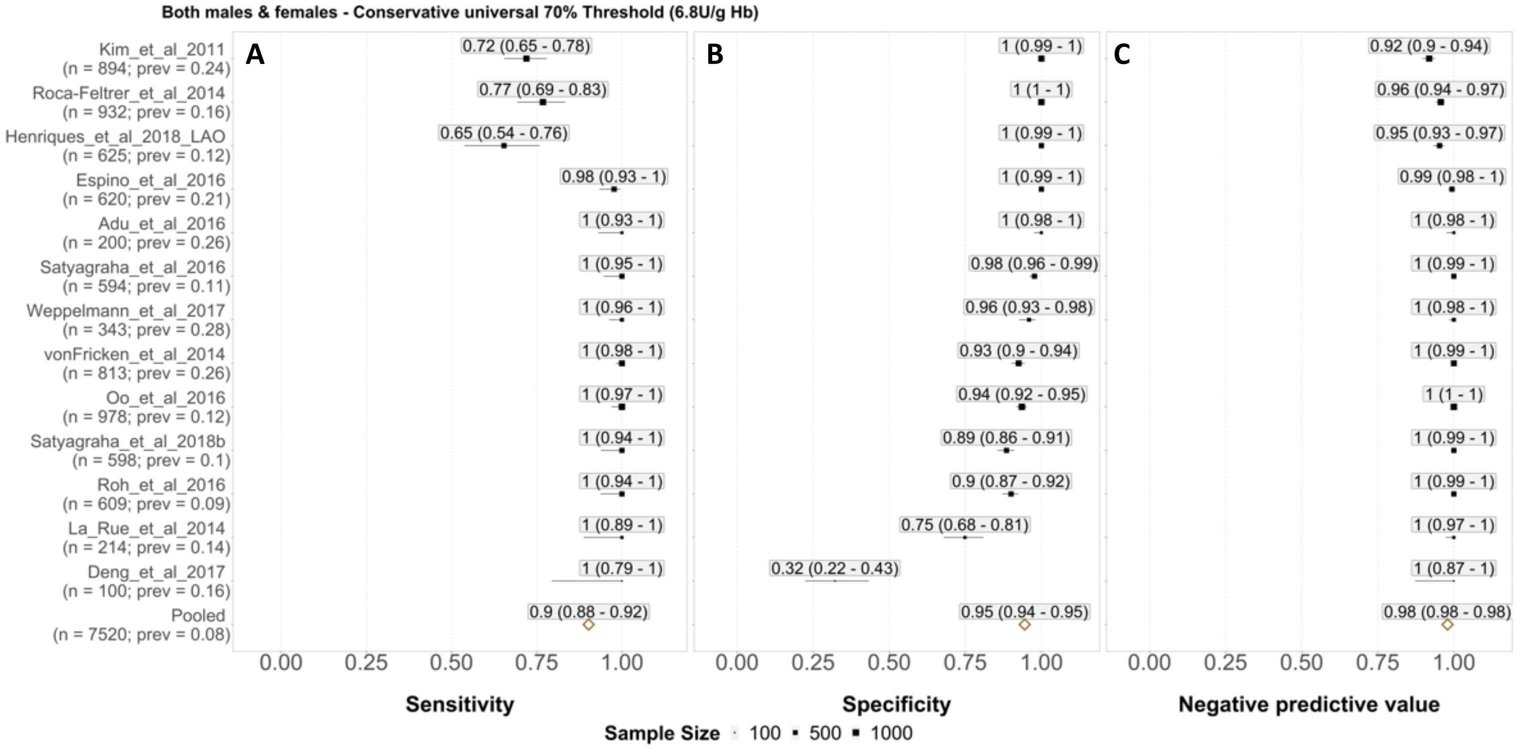

S16 Fig. Study-wise diagnostic performance of a conservative universal 70% enzyme activity threshold - both males and females. Study-wise sensitivity (**Panel A**), specificity (**Panel B**), & negative predictive value (**Panel C**) were calculated with 'exact' binomial confidence limits using site specific local AMM as the reference. The universal 70% threshold used corresponds to 6.8 U/g Hb, as derived from a conservative universal AMM (9.7 U/g Hb) – see methods for definition. Sample size and prevalence of severe or intermediate G6PD deficiency (enzyme activity <70% local AMM) are indicated for each study on the y-axis. Points are sized according to sample size. Data were included from 7,520 males and females tested by Trinity.

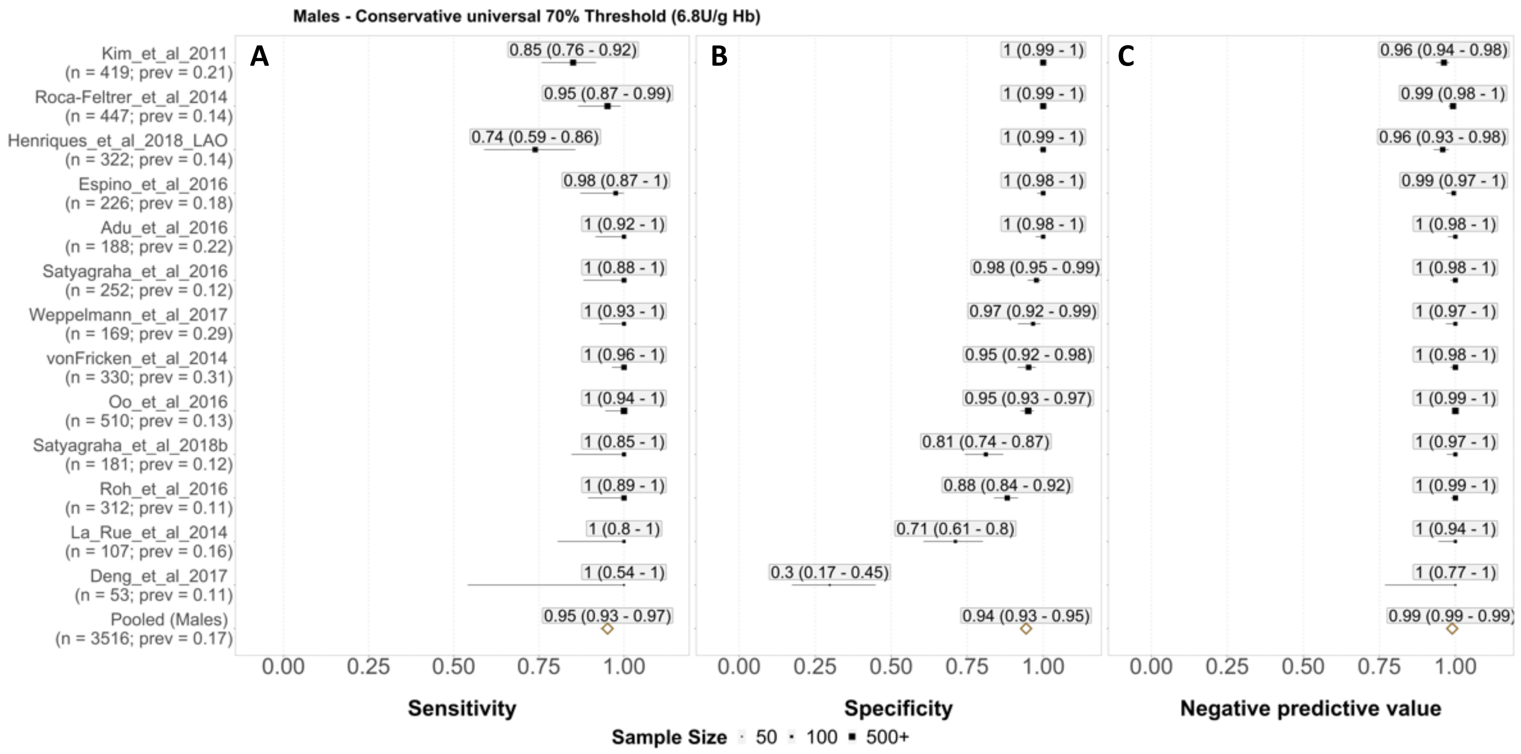

S17 Fig. Study-wise diagnostic performance of a conservative universal 70% enzyme activity threshold – males.
Study-wise sensitivity (**Panel A**), specificity (**Panel B**), & negative predictive value (**Panel C**) were calculated with 'exact' binomial confidence limits using site specific local AMM as the reference. The universal 70% threshold used corresponds to 6.8 U/g Hb, as derived from a conservative universal AMM (9.7 U/g Hb) – see methods for definition. Sample size and prevalence of severe or intermediate G6PD deficiency (enzyme activity <70% local AMM) are indicated for each study on the y-axis. Points are sized according to sample size. Data were included from 3,516 males tested by Trinity.

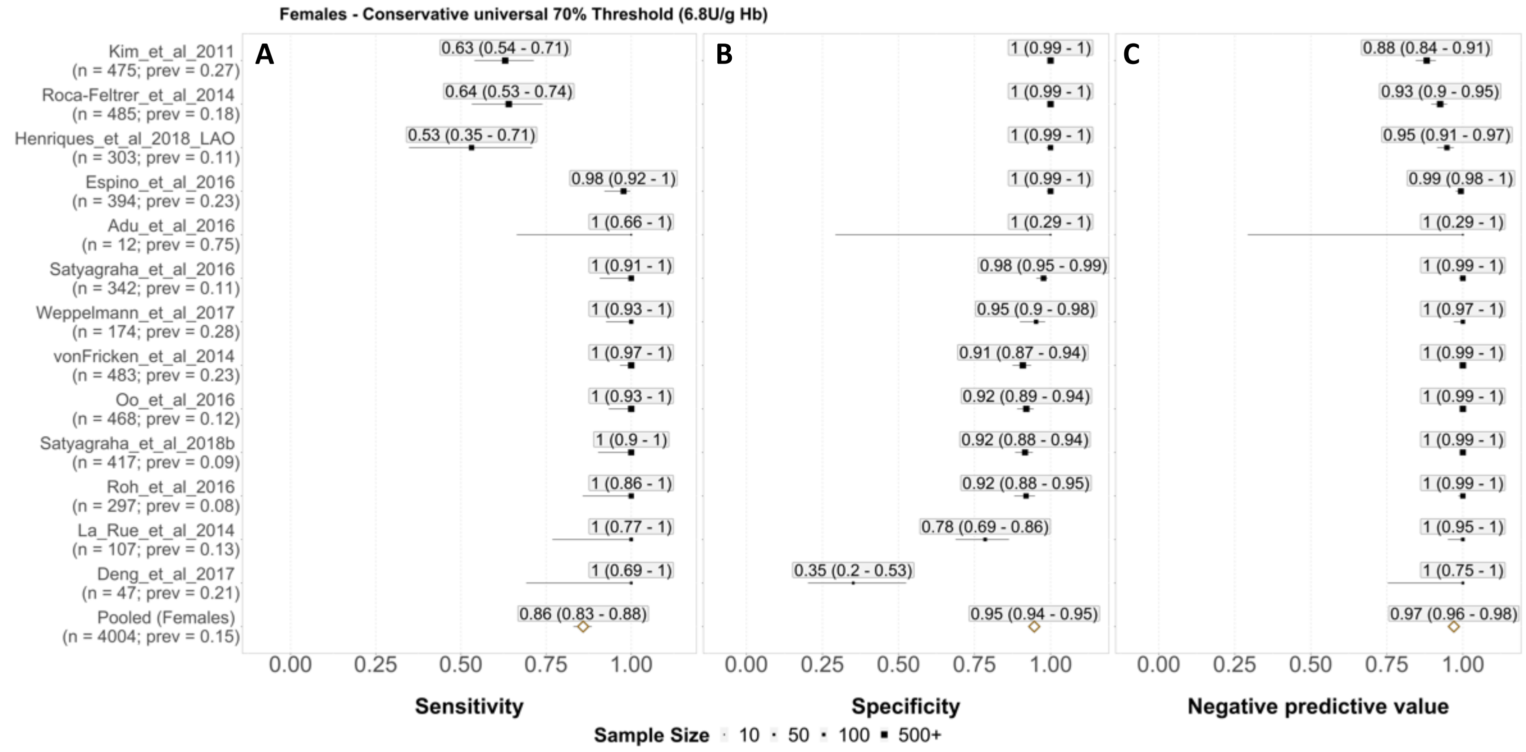

S18 Fig. Study-wise diagnostic performance of a conservative universal 70% enzyme activity threshold – females.
Study-wise sensitivity (**Panel A**), specificity (**Panel B**), & negative predictive value (**Panel C**) were calculated with 'exact' binomial confidence limits using site specific local AMM as the reference. The universal 70% threshold used corresponds to 6.8 U/g Hb, as derived from a conservative universal AMM (9.7 U/g Hb) – see methods for definition. Sample size and prevalence of severe or intermediate G6PD deficiency (enzyme activity <70% local AMM) are indicated for each study on the y-axis. Points are sized according to sample size. Data were included from 7,520 males and females tested by Trinity.

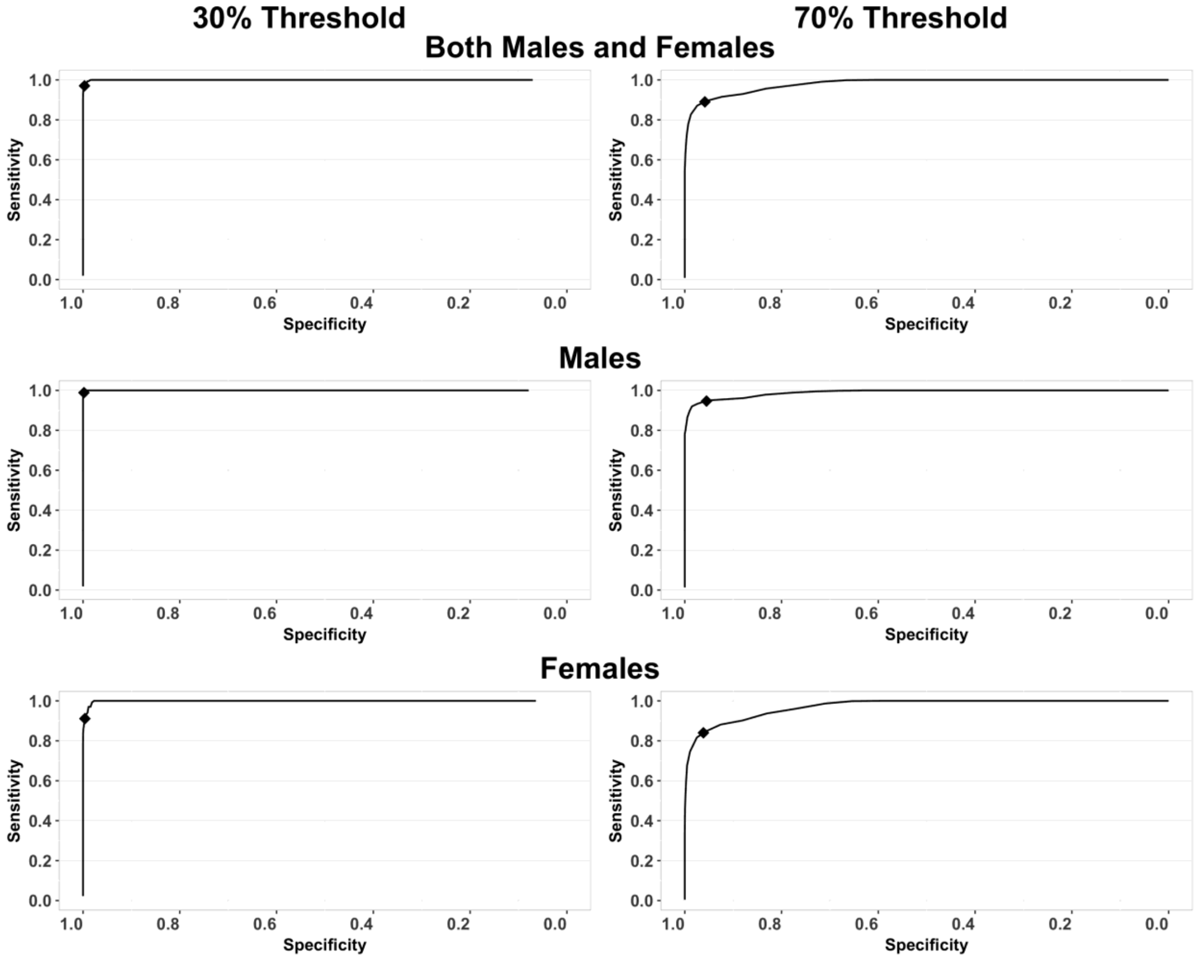

S19 Fig. Summary ROC Curves of the performance of universal enzyme activity thresholds.
Summary ROC curves were derived from the pooled database of G6PD activity measurements and depicted by sex (Males, Females, Both; columns) and diagnostic threshold (30%, 70%; columns). The location of the universal threshold used in this manuscript (100% = 9.4 U/g Hb) is depicted as a black dot on each panel.
