# Supplementary material for: Quantification of glucose-6-phosphate dehydrogenase activity by spectrophotometry: A systematic review and meta-analysis
Source: PLoS Med. 2020 May 14;17(5):e1003084. doi: 10.1371/journal.pmed.1003084 (PMC7224463; doi:10.1371/journal.pmed.1003084)
Supplement: S4 File — (PDF) [file pmed.1003084.s004.pdf]

S5 Table. List of data contacts for each included study.

Source Name	Source Contact	Citation
Adu et al, 2016	Patrick Adu <patrick.adu@ucc.edu.gh>	Adu P, Simpong DL, Takyi G, Ephraim RK. Glucose-6-phosphate dehydrogenase deficiency and sickle cell trait among prospective blood donors: A cross-sectional study in Berekum, Ghana. Adv Hematol. 2016;2016:7302912. Epub 2016/10/06. doi: 10.1155/2016/7302912. PubMed PMID: 27703480; PubMed Central PMCID: PMCPMC5039272.
Bancone et al, 2015	Germana Bancone <Germana@tropmedres.ac>	Bancone G, Chu CS, Chowwiwat N, Somsakchaicharoen R, Wilaisrisak P, Charunwatthana P, et al. Suitability of capillary blood for quantitative assessment of G6PD activity and performances of G6PD point-of-care tests. Am J Trop Med Hyg. 2015;92(4):818-24. Epub 2015/02/04. doi: 10.4269/ajtmh.14-0696. PubMed PMID: 25646252; PubMed Central PMCID: PMCPMC4385780.
Brito et al, 2016	Marcus Lacerda <marcuslacerda.br@gmail.com>; Wuelton Marcelo <wueltonmm@gmail.com>	Brito MAM, Peixoto HM, Almeida ACGd, Oliveira MRFd, Romero GAS, Moura-Neto JP, et al. Validation of the rapid test Carestart G6PD among malaria vivax-infected subjects in the Brazilian Amazon. Rev Soc Bras Med Trop. 2016;49(4):446-55.
Charoenkwan et al, 2014	Pimlak Charoenkwan <pimlak.c@cmu.ac.th>	Charoenkwan P, Tantiprabha W, Sirichotiyakul S, Phusua A, Sanguansermsri T. Prevalence and molecular characterization of glucose-6-phosphate dehydrogenase deficiency in northern Thailand. Southeast Asian J of Trop Med Public Health. 2014;45(1):187-93. Epub 2014/06/27. PubMed PMID: 24964669.
Deng et al, 2017	Liwang Cui <luc2@psu.edu>; Yongshu He <yongshuhe@hotmail.com>	Deng Z, Yang F, Bai Y, He L, Li Q, Wu Y, et al. Co-inheritance of glucose-6-phosphate dehydrogenase deficiency mutations and hemoglobin E in a Kachin population in a malaria-endemic region of Southeast Asia. PLoS ONE. 2017;12(5):e0177917. doi: 10.1371/journal.pone.0177917.
Egesie et al, 2008	Julie Egesie <juliegesie@yahoo.com>	Egesie OJ, Joseph DE, Isiguzoro I, Egesie UG. Glucose-6-phosphate dehydrogenase (G6PD) activity and deficiency in a population of Nigerian males resident in Jos. Niger J Physiol Sci. 2008;23(1-2):9-11. Epub 2009/05/13. PubMed PMID: 19434206.
Espino et al, 2016	Effie Espino <fe.espino2012@gmail.com>	Espino FE, Bibit J-A, Sornillo JB, Tan A, von Seidlein L, Ley B. Comparison of three screening test kits for G6PD enzyme deficiency: Implications for its use in the radical cure of vivax malaria in remote and resource-poor areas in the Philippines. PLoS ONE. 2016;11(2):e0148172. doi: 10.1371/journal.pone.0148172.
Hailu et al, 2018	Asrat Hailu <hailu_a2004@yahoo.com>	Unpublished study.

Hamid et al, 2018	mahdi@iend.org <mahdi@iend.org>	Hamid MMA, Thriemer K, Elobied ME, Mahgoub NS, Boshara SA, Elsafi HM, et al. Low risk of recurrence following artesunate–Sulphadoxine–pyrimethamine plus primaquine for uncomplicated Plasmodium falciparum and Plasmodium vivax infections in the Republic of the Sudan. Malar J. 2018;17(1):117.
Henriques et al, 2018 KHM	giselaclhenriques@gmail.com <giselaclhenriques@gmail.com>	Henriques G, Phommasone K, Tripura R, Peto TJ, Raut S, Snethlage C, et al. Comparison of glucose-6 phosphate dehydrogenase status by fluorescent spot test and rapid diagnostic test in Lao PDR and Cambodia. Malar J. 2018;17(1):243. doi: 10.1186/s12936-018-2390-6.
Henriques et al, 2018 LAO	giselaclhenriques@gmail.com <giselaclhenriques@gmail.com>	Henriques G, Phommasone K, Tripura R, Peto TJ, Raut S, Snethlage C, et al. Comparison of glucose-6 phosphate dehydrogenase status by fluorescent spot test and rapid diagnostic test in Lao PDR and Cambodia. Malar J. 2018;17(1):243. doi: 10.1186/s12936-018-2390-6.
Khim et al, 2013	Didier MENARD <didier.menard@pasteur.fr>	Khim N, Benedet C, Kim S, Kheng S, Siv S, Leang R, et al. G6PD deficiency in Plasmodium falciparum and Plasmodium vivax malaria-infected Cambodian patients. Malar J. 2013;12(1):171. doi: 10.1186/1475-2875-12-171.
Kim et al, 2011	Didier MENARD <didier.menard@pasteur.fr>	Kim S, Nguon C, Guillard B, Duong S, Chy S, Sum S, et al. Performance of the CareStart™ G6PD deficiency screening test, a point-of-care diagnostic for primaquine therapy screening. PLoS ONE. 2011;6(12):e28357. doi: 10.1371/journal.pone.0028357.
LaRue et al, 2014	Domingo, Gonzalo <gdomingo@path.org>	LaRue N, Kahn M, Murray M, Leader BT, Bansil P, McGray S, et al. Comparison of quantitative and qualitative tests for glucose-6-phosphate dehydrogenase deficiency. Am J Trop Med Hyg. 2014;91(4):854-61. doi: 10.4269/ajtmh.14-0194. PubMed PMID: PMC4183416.
Ley et al, 2016	Benedikt Ley <Benedikt.Ley@menzies.edu.au>	Ley B, Alam MS, Thriemer K, Hossain MS, Kibria MG, Auburn S, et al. G6PD deficiency and antimalarial efficacy for uncomplicated malaria in Bangladesh: A prospective observational study. PLoS ONE. 2016;11(4):e0154015. doi: 10.1371/journal.pone.0154015.
Ley et al, 2017	Benedikt Ley <Benedikt.Ley@menzies.edu.au>	Ley B, Thriemer K, Jaswal J, Poirot E, Alam MS, Phru CS, et al. Barriers to routine G6PD testing prior to treatment with primaquine. Malar J. 2017;16(1):329. doi: 10.1186/s12936-017-1981-y.
Ley et al, 2018	Benedikt Ley <Benedikt.Ley@menzies.edu.au>	Unpublished study.
Lin et al, 2017	Chanthap Lon chanthapl.ca@afrims.org	Lin JT, Lon C, Spring MD, Sok S, Chann S, Ittiverakul M, et al. Single dose primaquine to reduce gametocyte carriage and Plasmodium falciparum transmission in Cambodia: An open-label randomized trial. PLoS ONE. 2017;12(6):e0168702. doi: 10.1371/journal.pone.0168702.

Oo et al, 2016	Germana Bancone <Germana@tropmedres.ac>	Oo NN, Bancone G, Maw LZ, Chowwiwat N, Bansil P, Domingo GJ, et al. Validation of G6PD point-of-care rests among healthy volunteers in Yangon, Myanmar. PLoS ONE. 2016;11(4):e0152304-e. doi: 10.1371/journal.pone.0152304. PubMed PMID: 27035821.
Pal et al, 2018	Domingo, Gonzalo <gdomingo@path.org>	Pal S, Bansil P, Bancone G, Hrutkay S, Kahn M, Gornsawun G, et al. Evaluation of a novel quantitative test for glucose-6-phosphate dehydrogenase deficiency: bringing quantitative testing for glucose-6-phosphate dehydrogenase deficiency closer to the patient. Am J Trop Med Hyg. 2018:-. doi: 10.4269/ajtmh.18-0612.
Palasuwan et al, 2018	Duangdao Palasuwan <nantadao@gmail.com>	Unpublished study.
Poespoprodjo et al, 2018	Jeanne Rini Poespoprodjo <didot2266@yahoo.com>	Unpublished study.
Roca-Feltrer et al, 2014	Didier MENARD <didier.menard@pasteur.fr>	Roca-Feltrer A, Khim N, Kim S, Chy S, Canier L, Kerleguer A, et al. Field trial evaluation of the performances of point-of-care tests for screening G6PD deficiency in Cambodia. PLoS ONE. 2014;9(12):e116143. doi: 10.1371/journal.pone.0116143.
Roh et al, 2016	Parikh, Sunil <sunil.parikh@yale.edu>	Roh ME, Oyet C, Orikiriza P, Wade M, Mwanga-Amumpaire J, Boum Y, 2nd, et al. Screening for glucose-6-phosphate dehydrogenase deficiency using three detection methods: A cross-sectional survey in Southwestern Uganda. Am J Trop Med Hyg. 2016;95(5):1094-9. doi: 10.4269/ajtmh.16-0552. PubMed PMID: 27672207.
Satyagraha et al, 2016	Ari W. Satyagraha <ari@eijkman.go.id>	Satyagraha AW, Sadhewa A, Elvira R, Elyazar I, Feriandika D, Antonjaya U, et al. Assessment of point-of-care diagnostics for G6PD deficiency in malaria endemic rural Eastern Indonesia. PLoS Negl Trop Dis. 2016;10(2):e0004457. doi: 10.1371/journal.pntd.0004457.
Satyagraha et al, 2018a	Ari W. Satyagraha <ari@eijkman.go.id>	Unpublished study.
Satyagraha et al, 2018b	Ari W. Satyagraha <ari@eijkman.go.id>	Unpublished study.
Shannon et al, 2015	Wasif Ali Khan <wakhan@icddrb.org>	Shannon KL, Ahmed S, Rahman H, Prue CS, Khyang J, Ram M, et al. Hemoglobin E and glucose-6-phosphate dehydrogenase deficiency and Plasmodium falciparum malaria in the Chittagong Hill Districts of Bangladesh. Am J Trop Med Hyg. 2015;93(2):281-6. doi: https://doi.org/10.4269/ajtmh.14-0623.
vonFricken et al, 2014	Michael E Von Fricken <mvonfric@gmu.edu>	von Fricken ME, Weppelmann TA, Eaton WT, Alam MT, Carter TE, Schick L, et al. Prevalence of glucose-6-phosphate dehydrogenase (G6PD) deficiency in the Ouest and Sud-Est departments of Haiti. Acta Trop. 2014;135:62-6. doi: https://doi.org/10.1016/j.actatropica.2014.03.011.

Weppelmann et al, 2017	Michael E Von Fricken <mvonfric@gmu.edu>	Weppelmann TA, von Fricken ME, Wilfong TD, Aguenza E, Philippe TT, Okech BA. Field trial of the CareStart biosensor analyzer for the determination of glucose-6-phosphate dehydrogenase activity in Haiti. Am J Trop Med Hyg. 2017;97(4):1262-70. Epub 2017/08/19. doi: 10.4269/ajtmh.16-0714. PubMed PMID: 28820691.
