# Supplementary figures and images for: Quantification of glucose-6-phosphate dehydrogenase activity by spectrophotometry: A systematic review and meta-analysis
Source: PLoS Med. 2020 May 14;17(5):e1003084. doi: 10.1371/journal.pmed.1003084 (PMC7224463; doi:10.1371/journal.pmed.1003084)

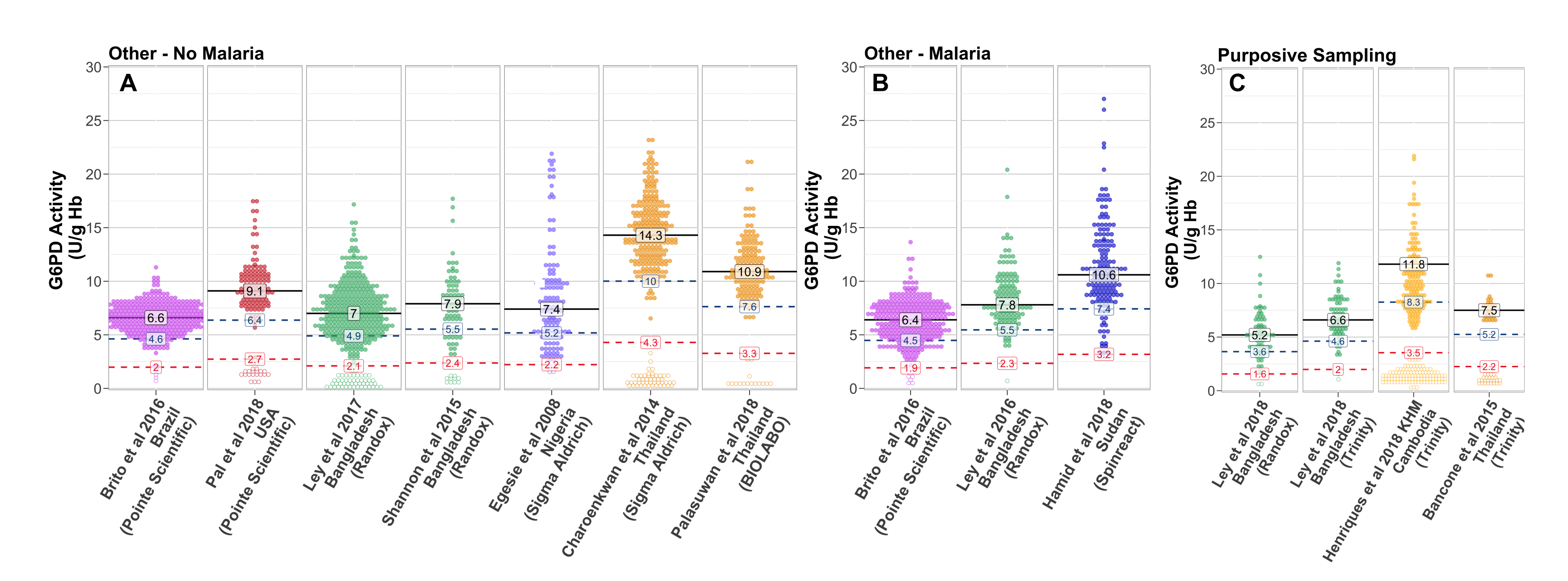

Supplement: S1 Fig — Comparison of G6PD activity distributions among males. (A, B) Studies using an assay other than Trinity, by malaria status, and (C) studies with purposive sampling. Assays used are indicated in brackets on the x-axis. The AMM (black text), 30% threshold (red), and 70% threshold (blue) are labelled for each study (in U/g Hb). Point colours indicate the country of origin of each study. Filled points represent G6PDn individuals (>30% study AMM), and hollow points indicate G6PDd individuals (<30% study AMM). Note: 100% G6PD activity shown for Henriques et al., 2018, and Bancone et al., 2015 (C), are as reported, not the AMM, due to strong oversampling of G6PDd individuals. Charoenkwan et al. [36] enrolled neonates, who may have had elevated G6PD activity [31, 32]. AMM, adjusted male median; G6PD, glucose-6-phosphate dehydrogenase; G6PDd, G6PD deficient; G6PDn, G6PD normal. (PNG) [file pmed.1003084.s005.png]
