# Supplementary material for: Quantification of glucose-6-phosphate dehydrogenase activity by spectrophotometry: A systematic review and meta-analysis
Source: PLoS Med. 2020 May 14;17(5):e1003084. doi: 10.1371/journal.pmed.1003084 (PMC7224463; doi:10.1371/journal.pmed.1003084)
Supplement: Attachment — Submitted filename: PMEDICINE-PaulEder.docx [file pmed.1003084.s010.docx]

PMEDICINE-D-19-02590_PaulEder

Quantification of glucose-6-phosphate dehydrogenase activity by spectrophotometry:

A systematic review and meta-analysis

Plasodium vivax infection is the predominant cause of malaria disease in sub-Saharan Africa, Southeast Asia, and parts of the Americas. It is well established that malaria prevalence from infection from P. vivax is complicated by the dormancy of the hypnozoite stage of the parasite in the liver. One class of drugs that can reach and kill the liver stage of these hiding hypnozoites is called the 8-aminoquinolines, of which tafenoquine is the newest to be approved. A single dose can be enough to lead to radical cure.

Paradoxically, although tafenoquine leads to a so-called radical cure with a single dose, the drug can cause dangerous hemolytic anemia in a portion of the malaria-infected population that cannot produce significant amounts of erythrocyte glucose-6-phosphate dehydrogenase (G6PD). As the G6PD gene is single copy and located on the X-chromosome, males can be hemizygous normal (G6PD activity > 95% of normal) or hemizygous deficient for the enzyme (< 30% of normal G6PD activity). The situation is more complicated for females, who can be homozygous normal (expressing > 95% of G6PD activity) or homozygous deficient (expressing < 30% of normal G6PD activity) or the most complicated group, heterozygous, where G6PD activity can be in the range of 30-70% of normal.

It is this last group that especially needs to be tested for G6PD activity and for which a universal cutoff is needed to determine whether recipients of the therapeutic are at risk of hemolytic anemia or not.

In desiring to move toward defining a universal threshold cutoff for patient intermediate enzyme activity and therefore low risk of hemolytic anemia in receiving tafenoquine, the authors wish to explore the diagnostic implications of applying this threshold by reviewing the consistency of enzyme activity across geographic regions and laboratories and even within laboratories for consistency among replicate testing.

Major Points:

The authors present a comprehensive data set of more than 15,000 blood specimens from 30 studies that had been winnowed from an initial review of 312 studies. This comprehensive review stratifies males and females to consider genetic effects, those infected and uninfected with malaria ahead of testing, broad geographic areas of testing, and includes six different quantitative laboratory instrumentation systems for G6PD testing. In total, these data support their claim that there is significant assay variation as well as laboratory variation in G6PD activity, which thus precludes the setting of a universal threshold or cutoff of G6PD activity to predict who would benefit and who is at risk for receiving tafenoquine for radical cure of malaria.

On the other hand, the authors multiple times hint tantalizingly that a handheld quantitative G6PD diagnostic device may allow greater standardization in the future without giving much detail on status or update of such a proposed device. Can it be developed on a simple platform and produced at scale and satisfy performance requirements? Can it be deployed in the communities in which it would be needed the most? What is the timeline for such a proposed product? These seem to be central to the issue that quantitative laboratory tests today are not reliable enough to have an absolute universal threshold for 70% activity assigned. A little more definitive detail on potential solutions in development rather than language that sounds hypothetical would strengthened the argument for the importance of the problem vis a vis the imminence of a potential solution.

Minor Points:

It is confusing to have the figure legends described in the body of the manuscript and the figures themselves lined up at the end of the manuscript. The two should go together for easier review.

In addition, some of the figure descriptions themselves are confusing: e.g., in the description for Table 2 and Fig. 2 described on p. 11, the text states that five studies were represented, for a total of 2204 sample data points, but the table and the figure each have six studies with 2296 total data points. If one study that contains 92 data points is dropped, then indeed that brings the totals to five studies and 2204 data points. Similarly for Fig. 3 on p. 12, the text says that there were nine studies used in this figure, seven using the Trinity device and three using the Randox device, making ten total, not nine. Closer attention in proofreading would have obviated these problems.

Also, it is not clear why the authors chose to have the y-axis for percent CV in Fig. 2B enumerated on a log scale. The data of %CVs had a range of only 1.5% to 15%. This could easily have been spread out on a linear scale. As is, all the data are compressed on the log scale just below and above the 10% line, thus not providing any insight to the reader.

Judgement:

With some re-doubling of proofreading efforts, this manuscript is sufficiently important for the audience of PLoS Medicine to merit publication. Moreover, the comprehensive dataset analyzed supports their claims of current automated test variation under a variety of conditions. I recommend that the manuscript be published.
