# Supplementary material for: Quantification of glucose-6-phosphate dehydrogenase activity by spectrophotometry: A systematic review and meta-analysis
Source: PLoS Med. 2020 May 14;17(5):e1003084. doi: 10.1371/journal.pmed.1003084 (PMC7224463; doi:10.1371/journal.pmed.1003084)
Supplement: Attachment — Submitted filename: Pfeffer_et_al_G6PDSpectro_Response.docx [file pmed.1003084.s011.docx]

Dr Thomas McBride

Senior Editor

*PLoS Medicine*

29^th^ October 2019

**Re.** **PMEDICINE-D-19-02311**

*Quantification of G6PD activity by spectrophotometry: a systematic review and meta-analysis*

Dear Dr McBride,

Thank you for your email regarding our manuscript and the opportunity to revise our submission. The reviews were favourable and acknowledged the importance of this study and the clinical implications of our findings, while raising a number of issues regarding the presentation and limitations of the study.

We have now addressed all of the reviewers’ and editorial comments, making revisions to the manuscript to clarify our methodology and its caveats, and highlight the implications of our findings. We have further processed our figures through PACE for formatting.

We believe that the revised manuscript is much improved for these changes. Robust quantitative diagnosis of G6PD activity will be crucial to ensuring patient safety following primaquine or tafenoquine administration and thus our study has important clinical implications for the radical cure of malaria. Our paper highlights the centrality of diagnostic thresholds within this context and calls for caution in applying standardized cut-offs across different laboratory and clinical contexts.

We hope that you would agree that the paper is now suitable for publication.

We are also aware of a complementary systematic review of the performance of the Accessbio/Carestart rapid diagnostic G6PD test that has just been accepted for publication by *PLoS Medicine* (PMEDICINE-D-19-02311R1), with several co-authors contributing to both papers. We wonder whether the editors would consider back to back publication to the raise the profile of this neglected issue that is so vital for the elimination of *P. vivax* malaria.

Thank you for your time and consideration.

Yours sincerely,

Daniel Pfeffer	Prof. Ric Price

**Line numbers refer to the marked-up manuscript**

**Editorial Requests:**

**1. PRISMA. Please replace the page numbers with paragraph numbers per section (e.g. "Methods, paragraph 1"), since the page numbers of the final published paper may be different from the page numbers in the current manuscript.**

Page numbers and line numbers have been replaced with paragraph numbers per section as requested.

**2. Thank you for agreeing to include the aggregate data in the supplementary files. However, I did not see a data file, please include this with your revision. Additionally, it would be helpful to include a supplementary text file listing the data contacts for each of the studies included in your analysis.**

S1 Table, the separate excel file, has been reformatted and the study-specific AMM’s added to represent an aggregated study-level data file.

An additional file S4File has been added to include contact details for all data contributors.

**3. In the Abstract Methods and findings, please provide the dates of search, data sources, types of study designs included, eligibility criteria, and synthesis/appraisal methods.**

This information has now been added to the Abstract (lines 103 - 112).

*“Individual-level data were pooled from studies that used G6PD spectrophotometry. Studies were identified via PubMed search (25/04/2018) and unpublished contributions from contacted authors (PROSPERO: CRD42019121414). Studies were excluded if they assessed only individuals with known haematological conditions, were family studies, or those for which insufficient details were available. Studies of patients with malaria were included but analysed separately from individuals without malaria. Included studies were assessed for risk of bias using an adapted form of the QUADAS-2 tool. Repeatability, and both intra- and inter-laboratory variability in G6PD activity measurements were compared between studies and pooled across the full dataset. A universal threshold for G6PD deficiency was derived and its diagnostic performance was compared to site-specific thresholds.”*

**4. In the last sentence of the Abstract Methods and Findings section, please describe the main limitation(s) of the study's methodology.**

A sentence on limitations has been added to the Abstract (lines 143 – 145).

*“Our ability to identify underlying factors associated with study-level heterogeneity was limited by the availability of covariate data and diverse study contexts and methodologies.”*

**5. Please reformat the Author Summary into 2-3 single sentence bullet points for each of the following questions. Bullet points should be objective, brief, succinct, specific, accurate, and avoid technical language.**

The author summary has been reformatted to fit this structure (lines 158 - 220).

**6. Please also note the addition of an additional author: Michele D. Spring** as discussed separately with the PLoS Medicine team via email.

**Reviewer #1**

We thank the reviewer for their positive and helpful response.

**1. A little more definitive detail on potential solutions in development rather than language that sounds hypothetical would strengthened the argument for the importance of the problem vis a vis the imminence of a potential solution.**

Thank you for the suggestion. We have now added additional details to the conclusion referring specifically to novel biosensor assays (Lines 598-606). Limited data are currently available from field-trials to confirm their superiority in this regard. Assessing comparability of measurements using these novel assays across different contexts will be a vital area for future research.

*“Novel point-of-care assays, such as recently developed quantitative biosensors, are currently being evaluated in field trials. These assays require less sample preparation and offer more robust performance across diverse temperature ranges and clinical contexts than spectrophotometry. Although such tests may provide superior inter-site comparability than the current reference method (spectrophotometry), until this has been demonstrated, routine quantitative diagnosis of G6PDd will require site- and assay-specific local definitions of G6PD activity to ensure that tafenoquine can be administered safely.”*

**2. It is confusing to have the figure legends described in the body of the manuscript and the figures themselves lined up at the end of the manuscript. The two should go together for easier review.**

To address this we have moved figure legends and inserted figures at the end of the manuscript (Lines 821-868) to make these easier to review.

**3. In addition, some of the figure descriptions themselves are confusing: e.g., in the description for Table 2 and Fig. 2 described on p. 11, the text states that five studies were represented, for a total of 2204 sample data points, but the table and the figure each have six studies with 2296 total data points. If one study that contains 92 data points is dropped, then indeed that brings the totals to five studies and 2204 data points. Similarly for Fig. 3 on p. 12, the text says that there were nine studies used in this figure, seven using the Trinity device and three using the Randox device, making ten total, not nine. Closer attention in proofreading would have obviated these problems.**

We agree that these were not clear. The confusion in Table 2/ Fig 2 and in Fig 3 has arisen from the fact that one study provided data from 92 of the same samples measured using two different assays (listed as separate lines in the table/figures).

We have now amended the table (Line 396) and figure legends (Lines 834-836; 846-847) to clarify this.

**4. Also, it is not clear why the authors chose to have the y-axis for percent CV in Fig. 2B enumerated on a log scale. The data of %CVs had a range of only 1.5% to 15%. This could easily have been spread out on a linear scale. As is, all the data are compressed on the log scale just below and above the 10% line, thus not providing any insight to the reader.**

We agree that a natural scale is easier to interpret and the figure has now been revised accordingly (Line 824).

N.B. the actual range of >99% of the CV values is from 0 to 50% (excluding 5 outliers outside of this range), the 1.5-15% values refer to the range of *mean* CV values across studies.

**Reviewer #2**

We thank the reviewer for their positive and helpful response.

**1. The main issue the authors should clarify is that it isn't clear how the authors pooled the AMM in the IPD and how they accounted for between study heterogeneity (starting 257). The authors are rather vague in their presentation of the statistical methods here: "A pooled universal AMM was calculated across a comparable subset of all included samples." Given the challenges in pooling medians across studies (non-normality assumed), I would presume the authors would have had to calculate a AMM across the entirety of the pooled sample directly from the pooled data in one-stage, and hence one of the limitations may in fact be not being able to account for between study-heterogeneity (as the dominant IPD methodology if pooling means would be to account for this in an error-term in the regression model). This would be an important consideration/limitation as that 75% of the samples come from Asia (Table 1) and study labs have large variations: the authors report range of difference from 0.65- 8.64 with coefficient of variation between 1.6% to 14.9%. Inter-replicate difference was significantly difference between studies. Despite showing that 5/6 assays were from Trinity assay - this still had high levels of variability.**

**This means that in Line 263 where a conservative threshold was derived as the upper bound of the 95% CI of the mean G6PD activity, the pooled mean G6DP activity should in fact take into consideration between study differences. As this can be derived from a pooled regression analyses, covariate adjustments, study heterogeneity can be incorporated into a hierarchical model either in one-stage of two-stages. It's unclear how the authors pooled the mean here as well and it would beneficial to clarify the methods.**

We apologise for the lack of clarity in the methods. In both cases the reviewer is correct in assuming that the pooled median and mean were derived directly from the pooled dataset without explicitly adjusting for study heterogeneity.

We chose this approach due to the limited availability of biologically plausible covariates (such as lab temperature, different technicians, technician training, reagent age/lot numbers etc.) that are likely responsible for observed study-level heterogeneity. Simultaneously, however, from a practical perspective, a pooled threshold would need to represent a single ‘treat’ or ‘don’t-treat’ cut-off across different contexts. Hence, if we had adjusted our pooled threshold with the covariates available, this would have meant that practitioners would also have to adjust their assay cut-offs based upon local factors – a level of complexity which is unlikely to be practical or offer utility beyond current practice.

As such the ‘naïve’ pooled thresholds here were ultimately chosen to quantify the implications of ignoring study-level heterogeneity in setting diagnostic thresholds. We have now revised the methods section (Lines 348-354; 361-363) to clarify this.

*“A universal AMM was calculated by applying the standard AMM formula to a comparable subset of all included samples. To control for differences due to assay variability or malaria status, the universal AMM was only derived from samples tested using the Trinity assay in patients without malaria. For each study separately, and across the pooled subset of data, the diagnostic performance (sensitivity, specificity and negative predictive value (NPV)) of this universal AMM was then evaluated at both the 30% and 70% thresholds, considering diagnoses derived from study-specific AMMs as the reference.”*

*“Due to the limited availability of relevant covariate data from included studies, the universal AMM, conservative threshold, and threshold performances were not adjusted for study-level covariates.”*

**2. Finally, it's nice to see the authors give an overall pooled sensitivity and specificity (Lines 365-367), but it's also not described in the methods how this was done and if study-level covariates were incorporated in these pooled sensitivity and specificity. For completeness of presentation, it would be also useful to present PPV, NPV at varying prevalence and derive an overall accuracy measure summary ROC plot.**

The overall sensitivity and specificity were not adjusted for study-level covariates for the same reasons as for the pooled AMM and mean stated in Comment #1 above.

We have now clarified this in the methods section (Lines 361-363).

*“Due to the limited availability of relevant covariate data from included studies, the universal AMM, conservative threshold, and threshold performances were not adjusted for study-level covariates.”*

For completeness, summary ROC plots have been also added as **S19 Fig** for each combination of sexes (both, males, females) and diagnostic threshold level (30%, 70%).

**3. Line 206: It's still not clear why the lower bound for was 2005 for laboratory changes and methodology. Was there some particular change around this time or was this just a pragmatic choice to limit the number of studies?**

The specific cut-off of 2005 does not reflect a particular change in methodology but is pragmatic. We had originally set out without a lower limit on search dates, but quickly found that almost none of the authors responding to our email requests still had access to older datasets. Furthermore, for those who did have the data this was rarely in a useable format or with appropriate quality control. Therefore, we restricted the analysis to datasets within the last 14 years for which availability of individual-level data was higher, and quality more easily comparable.

We have now revised the methods to clarify this (Lines 299-302).

*‘In view of the wide range of assay manufacturers and laboratory methodologies in the published literature, only papers published between January 2005 and April 2018 were screened for inclusion to ensure comparability of diagnostic protocols and quality control procedures.’*

**4. Line starting 243: The analysis uses AMM to define 100% G6PD activity, then uses WHO definitions to define categories for < 30% of the AMM is deficiency and activity between 30-70% for intermediate from tafenoquine clinical trials and not the recent WHO definition between 30-80% activity. It would be advantageous to benchmark also against the recent WHO definition as this should enhance the results policy implications (30-80% definition for intermediate activity)**

We agree that the divergence between the current WHO guidelines and defined cut offs for tafenoquine are confusing. The former reflects a historical perspective that was intrinsically conservative. There are now calls for the WHO to review these guidelines and it is likely that the 80% threshold will be lowered rather than the 70% threshold raised. Although we considered presenting both the 80% and 70% thresholds, we felt that this would complicate the results unnecessarily. We decided to follow the 70% threshold stated in the current Tafenoquine licensing by both the TGA in Australia and FDA in the USA which represents the most relevant cut offs that will determine clinical practice.

However, regardless of the specific threshold used, the primary message of the paper remains the same: a universal threshold at either 70% or 80% is inappropriate for spectrophotometry.”

**Reviewer #3**

We thank the reviewers for their positive and helpful comments.

**1. In light of the fact that universal testing of newborns for G6DP is being suggested and indeed implemented in some countries the important variation in sensitivity and specificity of the tests in newborns is an important area to explore further.**

We agree - this is a very important point. While newborn testing is recommended, and in practice in several countries, there is limited evidence regarding the performance of universal G6PD testing and how this varies with age. Indeed, unpublished data suggests that G6PD activity in an individual may even vary within acute malaria and subsequent convalescence. Unfortunately, the current dataset was not amenable for formal investigation of this issue, with very few studies enrolling newborns.

To address the reviewers’ concerns we acknowledge this issue in the discussion (Lines 576-578).

*“Furthermore, while it has been suggested that neonates have elevated G6PD activity [31, 32], introducing possible bias, few of the included studies enrolled newborns ([36, 37]).”*

Future insights from studies enrolling both newborns and adults across different contexts are needed to disentangle the effect of age from laboratory heterogeneity.

**2. Although patients with and without malaria were looked at it might have been interesting to know if other blood dyscrasias common in these populations such as thalassemia and sickle cell disease had an influence on the outcome of the tests if any**

We agree; this is an important issue that may influence the performance of G6PD measurement, particularly in areas with a strong historical selective pressure due to malaria. However, most studies included did not diagnose these disorders. Therefore we explicitly excluded any studies that exclusively recruited those with haematological conditions.

To acknowledge this limitation we have amended the final paragraph of the discussion (Lines 571-576):

*“First, although we excluded studies in which haematological conditions may have influenced observed G6PD activity levels, the final dataset consisted of samples from diverse clinical and community survey contexts. This may have led to unequal influence on the measured G6PD activity of undiagnosed health conditions such as other haematological conditions. However, there was no clear pattern in G6PD activity by study type.”*

**3. The use of the term gold standard seems a little ambitious as the standard cannot be a universal standard if the standard has to be set for the different assays and in different populations as seems to have to be the case. The intra-laboratory variation as well as the variation across different assay types requires that quality control is especially important and this is a very useful fact to emphasise especially in countries where quality control systems are weak.**

We agree. We followed convention in using the term ‘gold standard’ in this manuscript, but we acknowledge the reviewer’s hesitancy with this term. Nonetheless, despite its imperfections, it is widely used in the published literature as a comparator for gauging the accuracy of alternative novel diagnostics due to the resolution it provides relative to other phenotypic tests.

To address this, we have removed the term in some places where it was not necessary (e.g. Line 502 and used the term “reference method” in the author summary (Line 164) and conclusions (Line 604) to emphasize the need for more standardized approach to diagnosis.

*“Ultra-violet spectrophotometry is used as the reference test to measure G6PD activity, for validating new point-of-care diagnostics and to determine population-specific definitions of G6PD deficiency.*” *(Line 164)*

*“Novel point-of-care assays, such as recently developed quantitative biosensors, are currently being evaluated in field trials. These assays are designed to require less sample preparation and offer more robust performance across diverse temperature ranges and clinical contexts than spectrophotometry. As such, they may provide superior inter-site comparability than the current reference method (spectrophotometry), however, until this has been demonstrated, routine quantitative diagnosis of G6PDd will require site- and assay-specific local definitions of G6PD activity to ensure that tafenoquine can be administered safely. “ (Line 604)*

**4. When introducing the drugs in line 162 It might have been a good idea to explain the different ways these drugs are deployed ie Primaquine is used as a single low dose to deal with gametocytes of Pf which WHO says is safe for all levels of G6DP activity (although in countries with very weak pharmacovigilance systems this contention may not be completely true) Primaquine for 14 or 7 days (or over 8 weeks) for management of hypnozoites and tafenoquine which is a single dose and therefore carries more hazards and requires a higher degree of safety measures before administration.**

We agree that the clinical use of primaquine varies considerably between patients with *P. falciparum* malaria (single dose) and *P. vivax* malaria (14-day radical cure).

To address this, we have added the following sentence to the first paragraph of the introduction (Lines 241-246):

*“Primaquine is used to treat patients with malaria in two clinical scenarios: either as a single low dose to kill P. falciparum gametocytes and reduce transmission, or at a higher dose, usually administered over 14 days to kill P. vivax hypnozoites. Tafenoquine, is also an 8-aminoquinoline drug, but more slowly eliminated than primaquine. It has been licensed recently as a single dose hypnozoiticidal agent for P. vivax liver stages.”*

**5. When relying on qualitative tests ( those are the only ones universally available at the moment to National programmes the sensitivity and specificity of these tests against a locally derived gold standard is important Even when Primaquine is in the national protocol many health workers are reluctant to prescribe this drug due to perhaps exaggerated fears of Haemolysis. However good a test is it can never be perfect and so cases of haemolysis will occur and when training health workers and drug recipients it will be necessary to advise patient of how to detect haemolysis and provide access to transfusion services when needed. This will be even more important when tafenoquine is used as management of drug induced haemolysis will be even more challenging**

We agree. A key factor in this process is how the test performs in clinical practice and this involves factors beyond the sensitivity or specificity of the assay. Working towards maximising access to robust and practical diagnosis of G6PD deficiency, while minimizing haemolytic risk, is a key motivation of the current study.

To address this, we have added a sentence to the conclusions, (Lines 606-610):

*“No clinical diagnostic test is 100% reliable, hence guidelines for the implementation of radical cure treatment policies will need to be accompanied by education of patients and their carers, and training of health workers, so that the signs and symptoms of severe haemolysis can be detected early and clinical management revised accordingly.”*
