# Supplementary material for: Quantification of glucose-6-phosphate dehydrogenase activity by spectrophotometry: A systematic review and meta-analysis
Source: PLoS Med. 2020 May 14;17(5):e1003084. doi: 10.1371/journal.pmed.1003084 (PMC7224463; doi:10.1371/journal.pmed.1003084)
Supplement: Attachment — Submitted filename: Pfeffer_et_al_G6PDSpectro_Response.docx [file pmed.1003084.s012.docx]

**Requests from Editors:**

1. **To your abstract, please add summary demographic details for study participants.**

Summary details for age and sex have been added to the abstract with the following sentence (Line 110):

*“Participants were aged between 0 and 86 years, 44.4% (7,083 individuals) of whom were female.”*

2. **In your abstract and elsewhere, please add p values alongside 95% CI where available.**

P-values for statistical comparisons are included in brackets in both the abstract e.g. Line 117:

*“Assay precision varied between laboratories, as assessed by intra-laboratory variance in control measurements (from 0.1 to 1.5 U/g Hb; p<0.001) and study-wise mean coefficient of variation of replicate measures (from 1.6 to 14.9%; p<0.001).”*

and the main text, e.g. Line 330:

*“The percentage of samples that showed a high inter-replicate difference (>2 U/g Hb) differed significantly across all studies (p<0.001)…”*

3. **At line 122, would "... lack of availability of covariate data ..." be more appropriate?**

The text has been updated to include the words ‘lack of’ (line 126):

*“Our ability to identify underlying factors associated with study-level heterogeneity was limited by the lack of availability of covariate data and diverse study contexts and methodologies.”*

4. **At line 124, please begin the sentence "Our findings indicate that ..." or similar.**

This sentence has been updated accordingly (line 128):

*“Our findings indicate that there is substantial variation in G6PD measurements by spectrophotometry between sites.”*

5. **If appropriate, please adapt the wording at the start of the Methods section of your main text (around line 239 where you refer to the PROSPERO record) to note that the study was prospectively planned**

This text has been updated to include the term prospective (Line 238):

*“The data for this prospective meta-analysis were pooled from a systematic literature review and unpublished contributions from collaborating investigators (PROSPERO: CRD42019121414)…”*

6. **Please refer to the attached PRISMA checklist in the Methods section.**

Reference to PRISMA guidelines has been added to the first sentence of the methods section (line 240):

*“The data for this prospective meta-analysis were pooled from a systematic literature review and unpublished contributions from collaborating investigators (PROSPERO: CRD42019121414) according to PRISMA guidelines (S3 File).”*

7. **Towards the end of the Methods section, please add a sentence to note that specific ethics approval was not required for the present analysis, and if available the identity of the body responsible for that assessment.**

The following sentence has been added to the end of the methods section (Line 303):

*“Prior ethical approval was obtained for all primary studies included in the analysis.”*

8. **At line 305, please adapt the text "a total of ... sample data" to indicate whether this refers to number of individuals.**

This sentence has been updated accordingly to indicate that this refers to the number of individuals

(Line 310):

*“Data from 231 individuals were excluded for reasons stated in* ***Fig 1****.”*

9. **We suspect that the reference call-outs in table 2 should not be in superscript.**

The superscript has been removed from these citations in table 2.

10. **Please adapt reference call-outs to this style: "... endemic regions [3,4].".**

The citation style has been adapted to remove the extra space within the square brackets for in-text call-outs. For example (Line 195):

*“G6PD deficiency is a common inherited enzymopathy that is particularly prevalent in malaria-endemic regions [3,4].”*

11. **Please add full access details to reference 7.**

This reference has been updated accordingly.

**Academic Editor**

We thank the editor for their positive and helpful comments.

1. **I agree with previous comments of referees that the authors should not refer to spectrophotometry as the “Gold standard”; it is not, this is a notoriously difficult condition to measure, there are so many variants, and the functional significance, in relation to the extent of the haemolysis with with tafenoquine, is really not clear. Thus “reference standard” should be used throughout.**

We agree and have replaced the remaining uses of the term gold standard with ‘reference standard’.

Line 210: *“Ultra-violet (UV) spectrophotometry is the reference standard diagnostic method for quantifying G6PD enzyme activity.”*

Line 540: *“As such, they may provide superior inter-site comparability than the current reference standard of spectrophotometry, however, until this has been shown, routine quantitative diagnosis of G6PDd will require site- and assay-specific local definitions of G6PD activity to ensure that tafenoquine can be administered safely.”*

2. **The authors conclude that there is “substantial heterogeneity in G6PD measurements between sites…” I would encourage they describe this as “substantial variation in….”. The word heterogeneity implies that G6PD actually does vary sites but in fact it appears that it is the result largely of laboratory measures varying.**

We have replaced the word heterogeneity with ‘variation’ in both the abstract and discussion:

Line 128: *“Our findings indicate that there is substantial variation in G6PD measurements by spectrophotometry between sites.”*

Line 498: *“This variation demonstrates the importance of performing and monitoring replicate measurements and control sample measurements in order to minimize assay-based variability and maximize comparability of results. “*

3. **Under, what did the researchers find, third bullet, please try and avoid the use of the word “significant”. This is an ambiguous word, it can be qualitatively important, or it can mean some statistical evaluation using P 0.05. “Important” or “considerable” is more helpful.**

We agree that this word should have been avoided and have opted for ‘substantial’ instead (line 154):

*“There was substantial variation in the performance and absolute measurements of spectrophotometry conducted in different laboratories, hindering the definition of a universal cut-off for G6PD deficiency.”*

4. **On line 189, the authors don’t quite communicate to the reader that the concern with tafenoquine is the long half-life, and thus the persistence of the harmful haemolytic effect with this drug-it may be worse than PQ.**

We acknowledge that this concern could have been clearer and have adjusted this text to read (Line 190):

*“Tafenoquine, is another 8-aminoquinoline drug, which has recently been licensed as a single dose hypnozoiticidal agent for P. vivax liver stages. While well tolerated in the majority of recipients, standard dosing of either drug can cause severe haemolysis in patients with glucose-6-phosphate dehydrogenase (G6PD) deficiency [2]. Tafenoquine is more slowly eliminated than primaquine, hence patients are exposed to potentially haemolytic concentrations of the drug for longer. The successful roll-out of tafenoquine will therefore require more stringent G6PD screening than is currently available in malaria endemic areas.”*

**Reviewer #2**

1. **The authors mention that "****had we adjusted our pooled threshold with the covariates available, this would have meant that practitioners would also have to adjust their assay cut-offs based upon local factors - a level of complexity which is unlikely to be practical or offer utility beyond current practice." This is an interesting point and I thought warrants inclusion in the methodology to strengthen their rationale.**

We have added the following sentence to the methods section to strengthen this rationale

(Line 299):

*“**Furthermore, such adjustment for study-level covariates would impose an impractical level of complexity in clinical practice.”*

2. **I thank the authors for educating me on the WHO guidelines being out of date - I think this also is a nice point that should have a place in the discussion - The policy implications are that both thresholds being inadequate.**

We have added a few sentences to this effect to the discussion to highlight the fairly arbitrary nature of where the line between normal/intermediate deficiency is drawn (Lines 502-508).

*“**It is worth noting that diagnostic definitions of ‘intermediate’ G6PD activity have varied over time and contexts. The early ‘WHO classifications’ of G6PD variants considered anything above 60% to be ‘normal’ G6PD activity [36], current WHO guidelines place the cut-off at 80% normal activity [17], and prescription requirements for tafenoquine in the USA and Australia require a G6PD activity of 70% or higher [37]. Regardless of the definition, our study highlights the significant challenges in establishing an estimate of 100% G6PD activity (in clinically relevant units of U/g Hb) from which patients’ relative enzyme activity can be calculated.“*

**Production Issues**

FIGURES and GRAPHICS:

**1. & 2. Please ensure that all main figure files are cited in ascending numerical order in the main text of the article. Please ensure that all main figures are referenced in the manuscript as Fig 1, Fig 2, etc. (including capitalization), rather than Figure 1, Figure 2, etc. Please note, however, that the file names themselves must not include the space, i.e. Fig1.tif, Fig2.tif, etc.**

All figures are cited in ascending order as specified

3. **Please ensure that all main figure legends are placed after the paragraph containing their first in-text citation.**

Figure legends are included as specified.

TABLES and BOXES:

**1. Please ensure that all main tables in their entirety (including titles and footnotes), are placed after the paragraph containing their first in-text citation.**

All tables are included and cited in ascending order as specified

**2. Table 2 uses returns to control text wrapping. This is indicated by a [¶ or ↵] symbol when "Show Paragraph Marks" is turned on in Word. Please either split those cells into separate cells to achieve alignment or remove the returns.**

Tables 1-3 have all been reformatted to remove returns.

METADATA:

**1.** **Please ensure that the CRediT author contributions listed for each co-author are completed in full. To qualify for authorship, each author must meet at least one of the seven core contributions (conceptualization; data curation; formal analysis; investigation; methodology; software; validation), as well as at least one of the writing contributions (original draft preparation; review and editing). Authors may also be noted against any of the remaining contributions, however these alone will not qualify a contributor for authorship. Please ensure that the full contributions of each author are acknowledged in the "Add/Edit/Remove Authors" section of our submission form. If they do not meet criteria for authorship please let us know as soon as possible by emailing** [**plosmedicine@plos.org**](mailto:plosmedicine@plos.org)**.**

Author contributions have been reviewed and corrected where missing information.

**2. Please accurately complete the 'Competing Interests' section on our submission form, including any COIs declared by your co-authors. If you have no competing interests to declare, please add "The authors have declared that no competing interests exist". Otherwise please declare all competing interests beginning with the statement "I have read the journal's policy and the authors of this manuscript have the following competing interests:"**

This has been reviewed and amended.

4. **In accordance with PLOS' data policy, please ensure that your Data Availability Statement in the submission form clearly identifies how readers can access your data. Data cannot be available on request, or only accessible by contacting one of the authors. Note that your Data Availability Statement will be typeset as it is written, so please ensure it is in complete sentences and appears as you would like it to in the published version.**

The data availability statement has been reviewed and amended slightly to refer to S4 File.

ARTICLE FILE:

**1.** **Please remove the funding information from your manuscript file. The published version is pulled automatically from the “Financial Disclosure” field in the submission form and must not be duplicated. Please therefore take this opportunity to check that the information in this field is correct and completed in full.**

This has been removed from the file.

**2. Please remove the Competing Interests section from your manuscript file. The published version is pulled automatically from the "Competing Interests" field in the submission form and must not be duplicated. Please therefore take this opportunity to check that the information in this field is correct and completed in full.**

This has been removed from the file.

**3. Please remove your Author Contributions section from your manuscript file as this information is automatically pulled from the authors section of our submission form and must not be duplicated. Please therefore take this opportunity to check that the information in this field is correct and completed in full.**

This has been removed from the file.

5. **Please remove the data availability information from your manuscript file as this is automatically pulled from the "Data Availability" field in the submission form and must not be duplicated. Please therefore take this opportunity to check that the information in this field is correct and completed in full.**

This has been removed from the file.

REFERENCES:

**1. Please ensure your references are in the style of PLOS Medicine (ICMJE/Vancouver style). The reference style guide can be found here: [URL]. You can also find a downloadable EndNote file in these guidelines to assist you. Please do a manual check as well, as the EndNote file does not catch everything. Please note that the references will be typeset as they appear so please ensure the format is consistent.**

**2. Please put all reference citations in square brackets separated by either commas or dashes with no spaces, e.g. [1,2,3] or [1-3].**

**3. Please make sure all references are cited in ascending numerical order in the text.**

**4. PLOS' policy regarding references is that only published or accepted manuscripts should be included in the reference list. Papers that have been submitted but not yet accepted should not be cited.**

All of (1-4) above have been noted and the reference style has been reviewed to ensure citations adhere to these and in-text references updated to remove the extra space.

ADDITIONAL COMMENTS:

1. **Please quote the total number of participants (15,811) in the abstract, alongside the number of women. We suggest: "****Study participants (15,811) were aged between 0 and 86 years, and 44.4% (7,083) were women.**

This suggested text has been incorporated.

**2. Please remove the first author summary point; we suggest amending the second (now first) point to "Complete cure of Plasmodium vivax malaria, the most geographically widespread form of the disease, requires ...".**

This suggested text has been incorporated.

**3. At line 237, please make that "... data for this prospectively planned meta-analysis were ...".**

This suggested text has been incorporated.

ACKNOWLEDGMENTS:

**1. Please move your disclaimer to the end of the Acknowledgments section of your manuscript, with a line break between the main text of the acknowledgments and the disclaimer statement.**

This text has been moved to the acknowledgements section as requested.

REFERENCES:

**1. Please put all reference citations in square brackets separated by either commas or dashes with no spaces, e.g. [1,2,3] or [1-3].**

**2. Please make sure all references are cited in ascending numerical order in the text.**

All references remain in accordance with the above.

SUPPORTING INFORMATION:

**1. Please ensure that all Supporting Information files use the following format verbatim throughout the manuscript (including legends and all in-text citations): S1 Fig, S1 Table, S1 Text, S1 Data, etc. Each file type should start at 1 and increase numerically. Please note, however, that the file names themselves must use an underscore rather than a space, i.e. S1_Fig.tif, S1_Table.xlsx, S1_Text.doc, S1_Data.xlsx, S1_PRISMA_Checklist.docx, etc.**

**2. Please ensure that each supporting information file has a legend at the end of the manuscript file, after the Reference list.**

**3. Please ensure your supporting information files are individually uploaded to the File Inventory as file type 'Supporting Information' and are not present in the manuscript file.**

**4. Please upload your supporting information as individual files, and use the following naming format: S1 Text; S1 Fig; S1 Table, S1 Data, etc. Each file type should start as S1 and increase numerically: S1 Text, S2 Text, etc. Note that all relevant in-text citations and captions will need to be updated accordingly.**

To clarify references to supporting information. Explicit references to S3 File have been added in text	and S1 Fig has been removed from S3 File and included as a separate file.
